# A comparative analysis of nonhost resistance across the two *Triticeae* crop species wheat and barley

**DOI:** 10.1186/s12870-017-1178-0

**Published:** 2017-12-04

**Authors:** Rhoda Delventhal, Jeyaraman Rajaraman, Francesca L. Stefanato, Sajid Rehman, Reza Aghnoum, Graham R. D. McGrann, Marie Bolger, Björn Usadel, Pete E. Hedley, Lesley Boyd, Rients E. Niks, Patrick Schweizer, Ulrich Schaffrath

**Affiliations:** 1 Department of Plant Physiology, RWTH Aachen University, 52056 Aachen, Germany; 2 Leibniz-Institute of Plant Genetics and Crop Plant Research, 06466 Gatersleben, Germany; 3 Department of Disease and Stress Biology, John Innes Centre, Norwich Research Park, Colney Lane, Colney, Norwich, Norfolk, NR4 7UH UK; 4 Plant Breeding, Graduate School for Experimental Plant Sciences, Wageningen University & Research, Droevendaalsesteeg 1, 6708 PB Wageningen, the Netherlands; 5 Institute of Botany and Molecular Genetics, BioSC, RWTH Aachen University, 52056 Aachen, Germany; 6 The James Hutton Institute, Invergowrie, Dundee, Scotland DD2 5DA UK; 7 NIAB, Huntingdon Road, Cambridge, CB3 0LE UK; 8 Present address: Molecular microbiology, John Innes Centre, Norwich Research Park, Norwich, NR4 7UH UK; 9 Present address: Biodiversity and Integrated Gene Management Program (BIGM), International Center for Agriculture Research in the Dry Areas, Rabat, Morocco; 10 Present address: Seed and Plant Improvement Research Department, Khorasan Razavi Agricultural and Natural Resources Research and Education Center, AREEO, Mashhad, Iran

**Keywords:** Wheat, Barley, *Blumeria*, *Magnaporthe*, *Puccinia*, Adapted isolate, Non-adapted isolate, Nonhost resistance, Quantitative resistance, Global transcriptome analysis

## Abstract

**Background:**

Nonhost resistance (NHR) protects plants against a vast number of non-adapted pathogens which implicates a potential exploitation as source for novel disease resistance strategies. Aiming at a fundamental understanding of NHR a global analysis of transcriptome reprogramming in the economically important *Triticeae* cereals wheat and barley, comparing host and nonhost interactions in three major fungal pathosystems responsible for powdery mildew (*Blumeria graminis ff. ssp*.), cereal blast (*Magnaporthe sp*.) and leaf rust (*Puccinia sp*.) diseases, was performed.

**Results:**

In each pathosystem a significant transcriptome reprogramming by adapted- or non-adapted pathogen isolates was observed, with considerable overlap between *Blumeria, Magnaporthe* and *Puccinia.* Small subsets of these general pathogen-regulated genes were identified as differentially regulated between host and corresponding nonhost interactions, indicating a fine-tuning of the general pathogen response during the course of co-evolution. Additionally, the host- or nonhost-related responses were rather specific for each pair of adapted and non-adapted isolates, indicating that the nonhost resistance-related responses were to a great extent pathosystem-specific. This pathosystem-specific reprogramming may reflect different resistance mechanisms operating against non-adapted pathogens with different lifestyles, or equally, different co-option of the hosts by the adapted isolates to create an optimal environment for infection. To compare the transcriptional reprogramming between wheat and barley, putative orthologues were identified. Within the wheat and barley general pathogen-regulated genes, temporal expression profiles of orthologues looked similar, indicating conserved general responses in *Triticeae* against fungal attack. However, the comparison of orthologues differentially expressed between host and nonhost interactions revealed fewer commonalities between wheat and barley, but rather suggested different host or nonhost responses in the two cereal species.

**Conclusions:**

Taken together, our results suggest independent co-evolutionary forces acting on host pathosystems mirrored by barley- or wheat-specific nonhost responses. As a result of evolutionary processes, at least for the pathosystems investigated, NHR appears to rely on rather specific plant responses.

**Electronic supplementary material:**

The online version of this article (10.1186/s12870-017-1178-0) contains supplementary material, which is available to authorized users.

## Background

The *Triticeae* include some of our most important cereal crops, with wheat (*Triticum aestivum*) and barley (*Hordeum vulgare*) accounting for almost one third of the world’s cereal production [1, 2]. With pests and diseases being responsible for 28% of wheat losses, resistance breeding is of great importance for food security [3–5]. Disease resistance may rely on preformed or inducible defense mechanisms, the latter requiring plant recognition of conserved pathogen-associated molecular patterns (PAMPs) and/or specific effectors that the pathogen has evolved to suppress plant defense or induce susceptibility [6, 7]. In host interactions basal resistance is regarded as part of the PAMP-Triggered Immunity (PTI) response, and if not suppressed by effectors can remain effective against an adapted pathogen as a quantitative, but relatively stable resistance [7, 8]. In contrast, Effector-Triggered Immunity (ETI) is a qualitative resistance, usually based on single major resistance (*R*-) genes acting upon effector, in this case termed avirulence factor, recognition. Although conferring complete immunity, *R*-gene resistance can be broken relatively easily by pathogen variants expressing mutated target effectors (virulence factor) [9].

Nonhost resistance (NHR), which by definition protects all individuals (genotypes) of a plant species against all isolates of a would-be pathogen, is seen as a novel source of durable and broad spectrum resistance [10–12]. In most interactions between plants and non-adapted pathogens, resistance has been shown to rely upon several genes that quantitatively contribute to NHR, e.g. NHR in barley to the wheat leaf rust (*Puccinia triticina; Pt*) and the wheat powdery mildew (*Blumeria graminis* f. sp. *tritici; Bgt*) pathogens [13–15]. However, cases of Mendelian NHR, conferred by a single gene, have also been reported, e.g. barley NHR to the maize pathogen *Cochliobolus carbonum* [16, 17].

Despite its potential impact on food security, the molecular frameworks underlying polygenic NHR in the *Triticeae* are poorly defined. It has been proposed that NHR might either be associated with PTI, whose mechanisms would suffice to defend against the non-adapted pathogen, or might involve stacked *R*-genes supporting each other by functional redundancy [18]. Unsolved questions in understanding NHR, which have driven the current study, include (i) the overlap of NHR with host resistance, (ii) whether the same NHR genes and mechanisms are effective against different pathogens, and (iii) whether NHR genes and mechanisms are conserved across different species within the *Triticeae*. The second question was to some extent addressed by Zellerhoff et al. [19] in a study comparing the transcriptional nonhost responses of barley to non-adapted isolates of the fungal pathogens *Blumeria* sp.*, Puccinia* sp. and *Magnaporthe* sp*.* The results of that study suggested that defense responses are in part pathosystem specific. However, to the best of our knowledge no direct comparison of NHR between species of the *Triticeae* has been reported.

A crucial aspect in studies of NHR is the choice of the plant-pathogen interactions, in particular with respect to the relationship between the nonhost and host plant species, and between the non-adapted and adapted pathogen isolates, as evolutionary proximity often is supposed to correlate with a larger overlap between host and nonhost defense responses [20]. For wheat and barley, pathosystems are available where adapted and non-adapted isolates are closely related, allowing a within pathosystem comparison of host and nonhost interactions, and a comparison of host and nonhost interactions between wheat and barley. Such pathosystems include the causal agents of the economically important diseases of powdery mildew (*Blumeria graminis),* cereal blast *(Magnaporthe sp.)* and cereal rust (*Puccinia sp.).* While rusts and powdery mildews have been important field diseases of both wheat and barley since domestication of these cereal crops, wheat blast has emerged as a field disease only since 1985, and is now present in South America and Asia [21–23].

These three pathosystems have distinct lifestyles, making them interesting subjects for a study of NHR. The obligate biotroph *Blumeria* only invades epidermal tissue, producing feeding structures, haustoria, within epidermal cells, and subsequently grows as an ectoparasite on the plant surface [24]. *Puccinia*, also an obligate biotroph, enters the plant through stomata, producing haustoria within mesophyll cells and grows mainly intercellularly [25]. In contrast, *Magnaporthe* isolates have a hemi-biotrophic lifestyle. They penetrate the plant epidermis forming invasive ‘haustoria-like’ hyphae in the initial biotrophic phase, then enter a necrotrophic phase, destroying the colonized epidermal and mesophyll tissue [26, 27]. Despite the different lifestyles, in all three pathosystems the first 48 h after spore germination are crucial for the outcome of the infection attempts. During this period the adapted isolates successfully invade the plant, while the growth of the non-adapted isolates is arrested, with plant defense responses occurring during or shortly after penetration [28–30].

In this study we investigate the early responses of wheat and barley to adapted and non-adapted isolates of the pathosystems *Blumeria sp., Magnaporthe sp.* and *Puccinia sp.* The study focused on the initial tissue colonization phase which is most crucial for initiation of effective defense responses. The later stages of host-colonization, in particular the reproductive stages, were excluded as the non-adapted isolates never reach these developmental stages. The objectives of this study were (i) to identify specific nonhost responses from general pathogen-regulated plant responses, (ii) to examine commonalities in nonhost responses between the three pathosystems and (iii) to compare nonhost-related responses between wheat and barley. Importantly, our results point to a high level of specificity of nonhost responses, both for pathosystems and for plant species.

## Results

A major goal of our study was the examination of transcriptional reprogramming in wheat and barley towards three fungal pathosystems *(Blumeria, Magnaporthe* and *Puccinia)*, comparing responses to adapted (host interactions) and non-adapted (nonhost interaction) isolates. To enable comparability we selected adapted isolates that displayed virulence in the host interaction and non-adapted isolates that evoked a rapid, cell-autonomous immune response in the nonhost situation on both wheat and barley. All experiments were conducted on the wheat cultivar Renan and the barley cultivar Vada.

### Infection phenotypes on wheat and barley in host and nonhost interactions

**Fig. 1:**
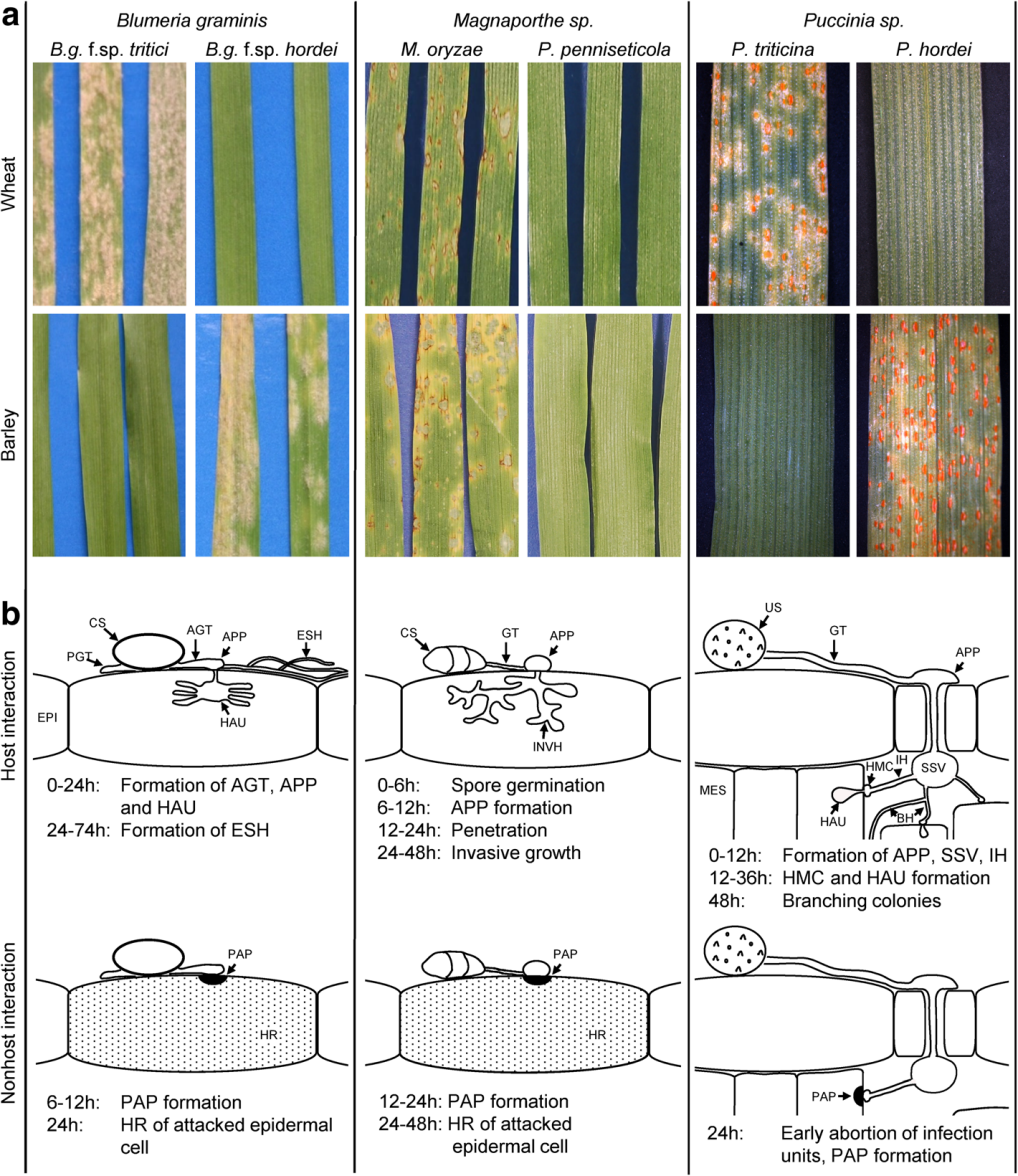
Phenotypes of host and nonhost interactions of wheat and barley with *Blumeria*, *Magnaporthe* and *Puccinia*. **a**, Macroscopic phenotypes of wheat cv. Renan and barley cv. Vada 10 days after inoculation with *Blumeria* isolates, 7 days after inoculation with *Magnaporthe* isolates and 20 days after inoculation with *Puccinia* isolates. **b**, Cytological phenotypes of host and nonhost interactions (summarized and simplified from Additional files 1, 2 and 3). Upper charts show typical fungal infection structures and their time of appearance as observed in host interactions. Lower charts show developmental stages in which fungal growth is typically arrested (and plant responses involved) in respective nonhost interactions. AGT: appressorial germ tube, APP: appressorium, BH: branching hyphae, CS: conidiospore, EPI: epidermis, ESH: elongating secondary hyphae, GT: germ tube, HAU: haustorium, HMC: haustorial mother cell, HR: hypersensitive response, IH: infection hypha, INVH: invasive hyphae, MES: mesophyll, PAP: papilla, PGT: primary germ tube, US: urediniospore, SSV: substomatal vesicle

The wheat and barley isolates causing powdery mildew (*Bgt* and *B. graminis* f. sp. *hordei, Bgh)* and the isolates causing leaf rust (*Pt,* and *P. hordei, Ph)* were fully virulent on their respective host plant species, but showed no macroscopic symptoms on the nonhost species (Fig. 1a). This absence of lesions was previously defined as type I NHR [6]. The *Magnaporthe* pathosystem (anamorph: *Pyricularia*) was represented by the adapted *M. oryzae (Mo)* isolates Br116.5 and TH6772, which caused a similar disease phenotype on wheat and barley, respectively (Fig. 1a). An isolate of the closely related *Magnaporthe* species, *Pyricularia penniseticola* (*Pp,* CD180) [31]*,* isolated from *Pennisetum sp.*, was used as the non-adapted *Magnaporthe* isolate for both wheat and barley, and produced no visible disease symptoms on either cereal species (Fig. 1a).

To identify at which stage in development (Fig. 1b) the non-adapted isolate ceased growth in the nonhost interaction, all interactions were investigated by microscopy at selected time points after inoculation (Additional file 1: Figure S1, Additional file 2: Figure S2, Additional file 3: Figure S3). In general, spores of all the fungal isolates were able to form germ tubes and appressoria on the leaf surface of both host and nonhost plants. This indicated that NHR did not operate at the pre-penetration stage, but rather became effective during penetration or post-penetration. While *Bgh* and *Bgt* had formed elongating secondary hyphae at 74 h past inoculation (hpi) in the host interactions, this developmental stage was never observed in the respective nonhost interactions (Additional file 1: Figure S1). Early plant responses included the accumulation of autofluorescent material underneath the appressorium or autofluoresence of the whole attacked epidermal cell, indicative of papilla-associated defense and initiation of hypersensitive response (HR), respectively [32, 33]. These reactions were observed at the majority of *Bgt* interaction sites, on wheat and barley, at 12 hpi, while in response to *Bgh* papillae or HR responses were first seen at 24 hpi (Additional file 1: Figure S1). This could indicate that *Bgt* is generally perceived faster than *Bgh,* and thus evokes an earlier plant reaction (irrespective of the host-status of the plant). However, the levels of epidermal HR were higher in the nonhost than in the host interactions at 24 hpi (Additional file 1: Figure S1) indicating a specific role of this defense response in NHR against *Blumeria.*

The blast isolates, *Mo* and *Pp*, had formed appressoria by 6–12 hpi in both the host and nonhost interactions (Additional file 2: Figure S2). The adapted *Mo* isolates Br116.5 and TH6772 were able to penetrate the epidermis of wheat and barley, respectively, invasive growth being first seen at 24 hpi. In contrast, the non-adapted *Pp* isolate CD180 was mostly arrested after appressorium formation (Additional file 2: Figure S2). While on wheat penetration attempts by CD180 failed completely, on barley invasive growth of CD180 was seen at a few interaction sites. Accordingly, the only autofluorescence response in wheat was seen associated with papilla formation, whereas barley responded to the non-adapted isolate with papillae as well as HR (Additional file 2: Figure S2). This implied that in wheat, at least in cv. Renan, penetration barriers might have been sufficient to arrest growth of CD180, while in the barley cv. Vada NHR operated at the penetration stage as well as post-penetration.

The rust fungi *Pt* and *Ph* entered through stomata and formed haustorial mother cells (HMC) in both host and nonhost interactions within 24 hpi (Additional file 3: Figure S3). *Ph* developed more rapidly than *Pt,* and had formed more infection hyphae and HMC at 12 hpi than *Pt* on both wheat and barley. Differences between host and nonhost interactions were first seen at 48 hpi, when the adapted isolates had formed haustoria and branching hyphae, while the non-adapted isolates were not able to colonize the mesophyll (Additional file 3: Figure S3).

### Reprogramming of the wheat and barley transcriptome in response to pathogen inoculation

Transcriptional analyses were performed on samples of inoculated and non- or mock-inoculated leaf tissues collected at four critical time points in the different pathosystems (Fig. 1b). As the entire *Blumeria* and the critical, early *Magnaporthe* interactions were confined to the cereal epidermis (Fig. 1b), tissue samples were taken only of the abaxial epidermis to optimize the concentration of transcripts impacted by the interactions. *Puccinia* interactions involved multiple leaf cell types (Fig. 1b), therefore whole leaves were sampled.

To identify genes differentially expressed in response to pathogen inoculation (both adapted or non-adapted isolates), inoculated and mock-inoculated samples were compared using the same statistical workflow for each pathosystem (Additional file 4: Table S1). These differentially expressed genes (DEGs) were subsequently referred to as the general pathogen-regulated genes. Overall, both wheat and barley reacted to pathogen inoculation with significant changes in transcript abundance of a great number of genes. In all pathosystems, except for barley-*Blumeria*, more DEGs were on average up-regulated than down-regulated after inoculation (Additional file 5: Figure S4). A considerable number of genes were regulated in common between the three pathosystems, in both wheat and barley, suggesting the existence of a biotic stress-related core transcriptome in the two cereals.

**Fig. 2:**
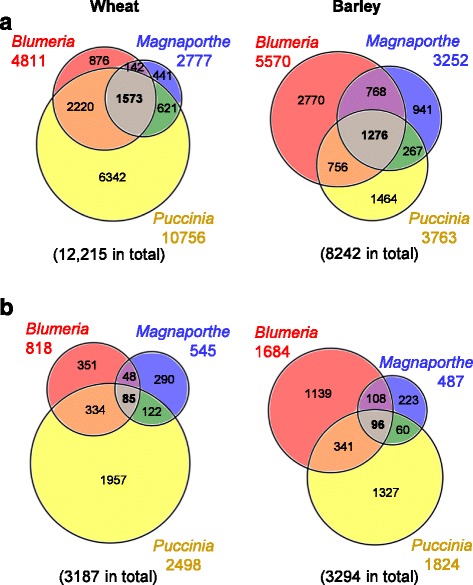
Identification of differentially expressed genes in wheat and barley for three pathosystems. **a**, The number of genes differentially expressed in wheat and barley for the three pathosystems *Blumeria, Magnaporthe* and *Puccinia* comparing inoculated samples (both adapted and non-adapted isolates) to mock inoculation. **b**, Those genes identified in (**a**) differentially expressed in wheat and barley for the three pathosystems *Blumeria, Magnaporthe* and *Puccinia* comparing samples inoculated with the adapted versus non-adapted isolates

In wheat, the number of DEGs was higher in the *Puccinia* pathosystem (10,756 DEGs) than in the *Blumeria* (4811 DEGs) or the *Magnaporthe* (2777 DEGs) pathosystems (Fig. 2a). Overall the combined number of wheat genes regulated in response to at least one of three pathogens was 12,215, which was taken as base for subsequent calculations. A high percentage of wheat DEGs (31%) were in common between the *Blumeria* and *Puccinia* pathosystem (Fig. 2a, orange and grey intersections), while 14 and 18% were in common between *Blumeria* and *Magnaporthe* (purple and grey), and *Magnaporthe* and *Puccinia* (green and grey), respectively (Fig. 2a). In wheat 13% of DEGs were shared between all three pathosystems (Fig. 2a, grey intersection). In barley, compared to wheat, similar numbers of DEGs were identified in the *Blumeria* (5570 DEGs) and the *Magnaporthe* (3252 DEGs) pathosystems, whereas fewer DEGs (3763) were found for the *Puccinia* pathosystem (Fig. 2a). Comparing the pathosystems, 25, 25 and 19% of the barley DEGs were shared between *Blumeria* and *Puccinia* (Fig. 2a, orange and grey), *Blumeria* and *Magnaporthe* (purple and grey), and *Magnaporthe* and *Puccinia* (green and grey), respectively (Fig. 2a). In barley, similar to wheat, 15% of DEGs were shared between all three pathosystems (Fig. 2a, grey intersection).

### Differential reprogramming of the wheat and barley transcriptome during host and nonhost interactions

To examine the differences between host and nonhost interactions in each pathosystem, DEGs were identified within the sets of general pathogen-regulated genes established above, by comparing samples from inoculations with adapted isolates to samples inoculated with non-adapted isolates (Additional file 6: Table S2). The number of genes differentially expressed between host and nonhost interactions were found to be less than 20% of the general pathogen-regulated genes for the pathosystems wheat-*Blumeria*, wheat-*Magnaporthe* and barley-*Magnaporthe*, and less than 50% for the other pathosystems (Fig. 2a and b). This indicated that the differential response of the plant transcriptome to adapted versus non-adapted isolates was relatively small, and that the general pathogen-induced reprogramming occurred rather irrespective of the isolate being adapted or non-adapted.

In both wheat and barley, the largest number of nonhost-related DEGs were identified in the *Puccinia* pathosystem, whereas the *Magnaporthe* pathosystem had the fewest nonhost-related DEGs (Fig. 2b). While 13% of DEGs were in common between the *Blumeria* and *Puccinia* pathosystems both in wheat and barley (Fig. 2b, orange and grey intersections), the other pathosystem comparisons revealed only 4–6% of shared DEGs (Fig. 2b). In both wheat and barley, the three pathosystems *Blumeria*, *Magnaporthe* and *Puccinia* had only 3% of the nonhost-related DEGs in common (Fig. 2b, grey intersections, Additional file 6: Table S2). Altogether, the nonhost-related DEGs showed only small overlaps between the different cereal-pathosystems (Fig. 2b) compared to the general pathogen-regulated genes (Fig. 2a).

### Conserved pathogen-induced transcriptional reprogramming between wheat and barley

To more directly interrogate the conservation of pathogen-induced transcriptional reprogramming between wheat and barley, putative gene orthologues had to be first identified. Two approaches were undertaken to identify the orthologues: (i) identification of reciprocal best blast hits in unigene sets of wheat and barley received from TaGI and HarvEST databases, respectively, and (ii) identification of orthologue clusters within the TaGI wheat unigenes and the IBSC barley coding sequences using the InParanoid tool [34] (Additional file 7: Table S3). In summary, 57.8 and 38.0% of the wheat and barley microarray probes could be linked by orthologue matching (Additional file 7: Table S3). We observed redundant linking of 37% of orthologue-assigned barley genes to more than one wheat orthologue, whereas only 7.1% of the orthologue-assigned wheat genes were assigned to more than one barley orthologue. In part, this redundancy might be attributed to the allo-hexaploid status of wheat, which would assign every barley gene to up to three wheat homoeologues.

**Fig. 3:**
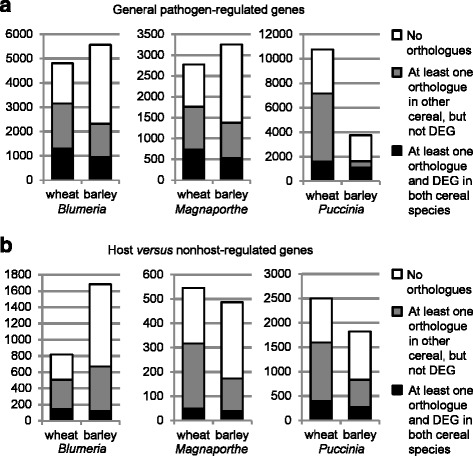
Comparison of differentially expressed genes identified in wheat and barley. Orthologous genes identified between wheat and barley were compared for each pathosystem, in both **a**, the general pathogen-regulated genes, and **b**, the host versus nonhost regulated genes. The total number of each gene list is shown as whole bars. For each list, the portion of genes that could be assigned to a putative orthologue is highlighted (grey and black) and comprises a subset whose orthologues were also present in the according gene list of the other cereal species (black). The black sections are represented in heat maps (Figs. 4, 5, 6 and 7)

To identify similarities in the transcriptional reprogramming in wheat and barley to pathogen inoculation the general pathogen-regulated DEGs were screened for matching orthologues (Fig. 3a). Out of the 4811 wheat DEGs found for the *Blumeria* pathosystem (Fig. 3a, whole bar) a total of 3152 DEGs had at least one orthologue in barley (Fig. 3a, grey and black sections), of which 1306 wheat DEGs had orthologues also present in the list of barley-*Blumeria* DEGs (Fig. 3a, black section). Conversely, of 5570 *Blumeria*-regulated barley DEGs, 2322 had orthologues in wheat, of which 946 had orthologues that were differentially regulated in the wheat-*Blumeria* pathosystem (Fig. 3a). In conclusion, 41% of the orthologue-assigned DEGs were general pathogen-regulated following *Blumeria* inoculation, in both wheat and barley. Similar percentages were found for the *Magnaporthe* pathosystem: 41% of the orthologue-assigned wheat DEGs and 39% of the orthologue-assigned barley DEGs had *Magnaporthe*-regulated orthologues in barley and wheat, respectively. In the *Puccinia* pathosystem, 23% of the orthologue-assigned wheat DEGs and 68% of the orthologue-assigned barley DEGs had *Puccinia*-regulated orthologues in the other cereal species. Taking into account that the number of DEGs found for the wheat-*Puccinia* interaction exceeded the number of barley-*Puccinia*-DEGs threefold (Fig. 2a), these percentages can be considered similar to those found for the other pathosystems. In each case the number of general pathogen regulated DEGs in the overlap of wheat and barley was larger than expected by chance (Additional file 8: Data S1). Taken together, the results indicated, that there exists a conserved general pathogen-regulated transcriptional response in wheat and barley.

**Fig. 4:**
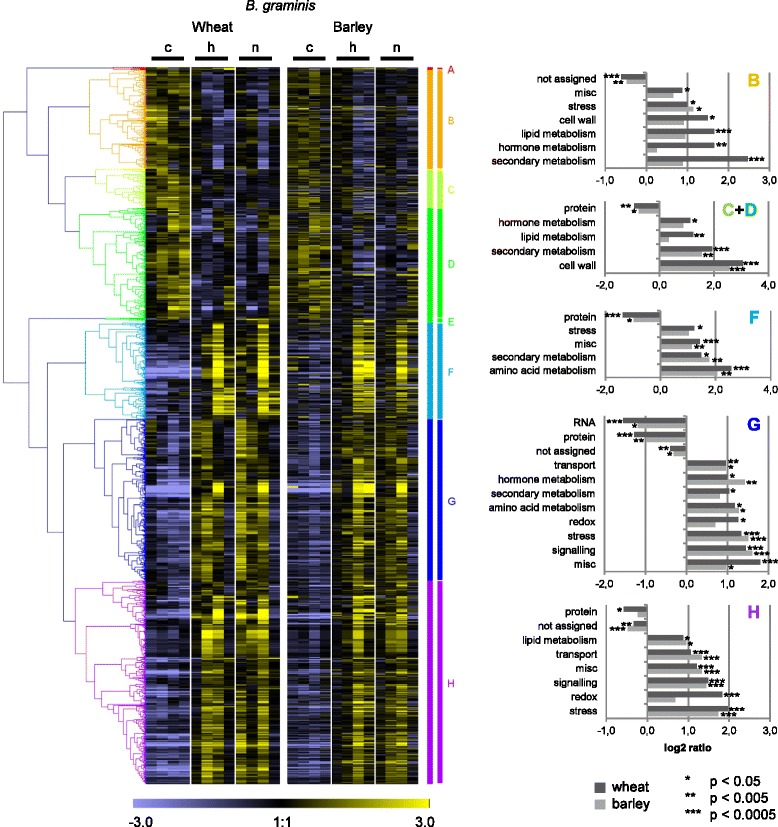
Heat maps of general pathogen-regulated orthologues in wheat and barley in *Blumeria* interactions. Differentially expressed genes (DEGs) of wheat in *Blumeria* interactions were filtered for assignment to DEGs of barley in *Blumeria* interactions. On the median-centered normalized signal intensities (c control, h: host interaction, n: nonhost interaction), a hierarchical clustering (Pearson correlation, average linkage) was performed with the software Genesis [88]. The median-centered signal intensities of barley orthologues were sorted accordingly and illustrated in a heat map. Clusters were named with letters and investigated for over- or underrepresentation of functional categories using MapMan ORA tool [35, 36]. All probes assigned to orthologues in the other species were taken as reference. The log ratio of presentation in the cluster and in the reference is given for BINs found to be significantly over- or underrepresented according to Fisher Exact Test (BINs comprising 5 or less probes of the cluster were excluded). misc.: miscellaneous

**Fig. 5:**
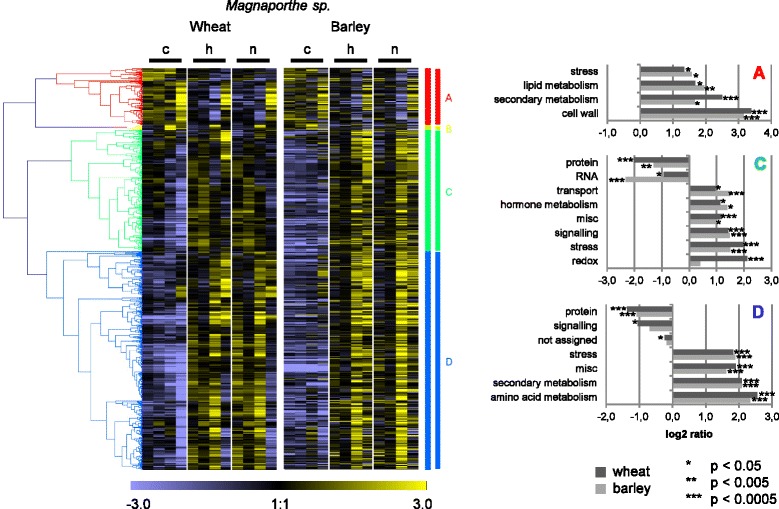
Heat maps of general pathogen-regulated orthologues in wheat and barley in *Magnaporthe* interactions. Differentially expressed genes (DEGs) of wheat in *Magnaporthe* interactions were filtered for assignment to DEGs of barley in *Magnaporthe* interactions. On the median-centered normalized signal intensities (c: control, h: host interaction, n: nonhost interaction), a hierarchical clustering (Pearson correlation, average linkage) was performed with the software Genesis [88]. The median-centered signal intensities of barley orthologues were sorted accordingly and illustrated in a heat map. Clusters were named with letters and investigated for over- or underrepresentation of functional categories using MapMan ORA tool [35, 36]. All probes assigned to orthologues in the other species were taken as reference. The log ratio of presentation in the cluster and in the reference is given for BINs found to be significantly over- or underrepresented according to Fisher Exact Test (BINs comprising 5 or less probes of the cluster were excluded). misc.: miscellaneous

**Fig. 6:**
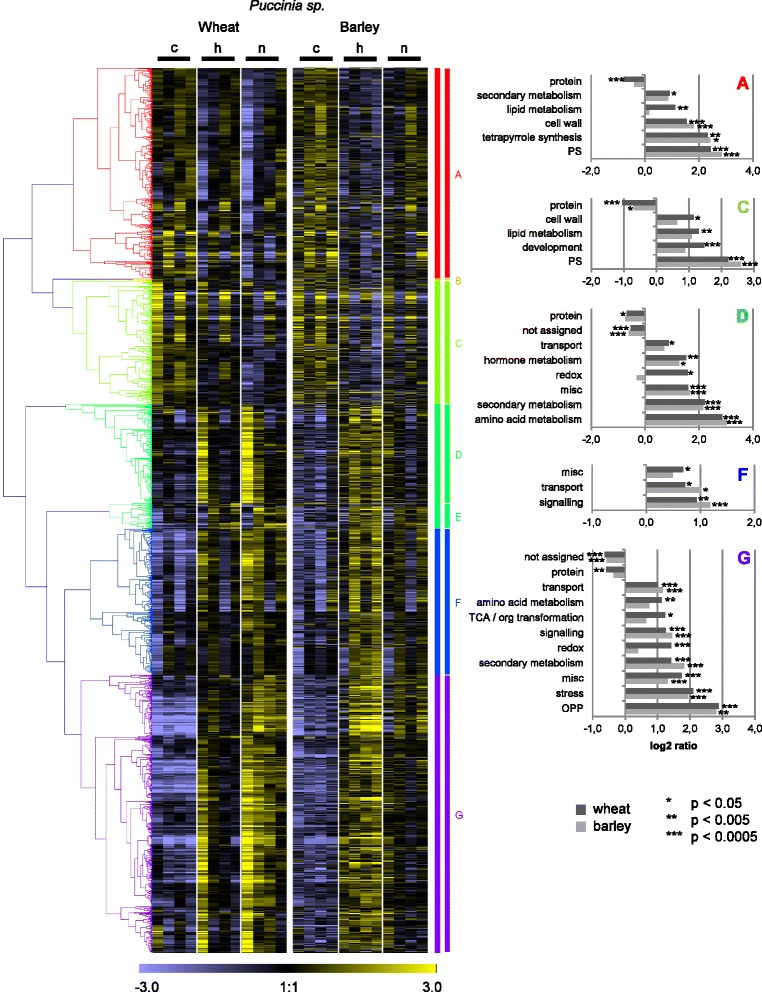
Heat maps of generally pathogen-regulated orthologues in wheat and barley in *Puccinia* interactions. Differentially expressed genes (DEGs) of wheat in *Puccinia* interactions were filtered for assignment to DEGs of barley in *Puccinia* interactions. On the median-centered normalized signal intensities (c: control, h: host interaction, n: nonhost interaction), a hierarchical clustering (Pearson correlation, average linkage) was performed with the software Genesis [88]. The median-centered signal intensities of barley orthologues were sorted accordingly and illustrated in a heat map. Clusters were named with letters and investigated for over- or underrepresentation of functional categories using MapMan ORA tool [35, 36]. All probes assigned to orthologues in the other species were taken as reference. The log ratio of presentation in the cluster and in the reference is given for BINs found to be significantly over- or underrepresented according to Fisher Exact Test (BINs comprising 5 or less probes of the cluster were excluded). PS, photosynthesis; misc.: miscellaneous

To examine the expression profiles of the orthologous general pathogen-regulated DEGs in wheat and barley over time heat maps were constructed (Figs. 4, 5, 6 and Additional file 9: Table S4). The wheat DEGs, for which orthologous barley DEGs had been identified, were clustered hierarchically according to their expression profiles in the *Blumeria*, *Magnaporthe* and *Puccinia* interactions, and the expression profiles of the corresponding barley orthologues placed alongside (Figs. 4, 5 and 6). In general, the wheat heat maps were very similar to the according barley heat maps, confirming that wheat and barley share a common pathogen-induced transcriptional response.

### Commonalities of the conserved transcriptional reprogramming in response to pathogens with different life-styles

To learn more about the function of the general pathogen-regulated gene orthologues, wheat and barley transcripts were assigned to functional categories using MapMan [35, 36]. Each gene cluster presented in Figs. 4, 5 and 6 was subjected to an over-representation analysis (ORA), pointing to those functional categories over- or under-represented within the gene set.

With the *Blumeria* pathosystem (Fig. 4), hierarchical clustering of the wheat genes and assignment of their barley orthologues resulted in clusters B, C and D being down-regulated, while clusters F, G and H were up-regulated after inoculation with adapted and non-adapted isolates (Fig. 4). The MapMan categories ‘secondary metabolism’, ‘hormone metabolism’ and ‘stress’ were significantly over-represented in several of these clusters. The category ‘cell wall’ was over-represented in the down-regulated clusters of genes (Fig. 4, clusters B, C, D), suggesting that down-regulation of normal cell wall metabolism is a common response in both wheat and barley to adapted and non-adapted *Blumeria* isolates, possibly allowing the formation of *de-novo* cell wall components by pathogen-induced gene products*.* The high importance for cell-wall re-construction for resistance to *Blumeria* has been shown [37–39]. The up-regulation of DEGs in cluster F clearly peaked at 24 hpi, in both host and nonhost interactions, in wheat and in barley (Fig. 4). In cluster F the functional categories ‘stress’, ‘secondary metabolism’, ‘amino acid metabolism’ and ‘miscellaneous enzyme families’ (including glucan endo-1,3-beta-glucosidases, cytochrome P450s, glutathione-S-transferases and peroxidases) were significantly over-represented. The results for ‘secondary metabolism’, ‘amino acid metabolism’ are in agreement with and extend reports on strong activation of the shikimate and phenylpropanoid pathways in *Blumeria*-attacked barley and wheat [40, 41]. In clusters G and H most wheat DEGs showed an earlier up-regulation in response to adapted and non-adapted *Blumeria* isolates (peaking at 6 or 12 hpi) than their corresponding barley orthologues (peaking at 12 or 24 hpi). In these clusters the category ‘protein’ was significantly under-represented, while ‘stress’, miscellaneous enzyme families’, ‘signaling’ and ‘transport’ were significantly over-represented (Fig. 4). This would be in agreement with reports on signaling and transport events being initiated during the early plant-pathogen infection stages [42]. Overall, despite the timing difference between wheat and barley mentioned for clusters G and H, the transcriptional regulation of the orthologues was highly conserved.

The functions of wheat and barley orthologous DEGs in the *Magnaporthe* pathosystem in many respects resembled those in the *Blumeria* pathosystem (Fig. 5). The transcription profiles in wheat and in barley looked very similar in adapted and non-adapted isolate interactions. Within the down-regulated wheat and barley DEG orthologues, the MapMan categories ‘lipid metabolism’, ‘stress’, ‘secondary metabolism’ and ‘cell wall’ were over-represented (Fig. 5, cluster A). As with the *Blumeria* pathosystem most up-regulated wheat DEGs responded earlier in response to *Magnaporthe* isolates than their corresponding barley orthologues (Fig. 5, cluster C). However, because only one cultivar per plant species was used, it remains unclear to what extent this difference reflects a species- or cultivar-specific effect. The corresponding DEGs showed a significant over-representation in categories ‘transport’ and ‘signaling’. The wheat genes up-regulated at 24 hpi in general had barley orthologues also up-regulated at 24 hpi, and showed over-representation in ‘amino acid metabolism’, ‘secondary metabolism’, ‘miscellaneous enzyme families’ and ‘stress’ (Fig. 5, cluster D). In both the *Blumeria* and *Magnaporthe* interactions at 24 hpi papillae and hypersensitive cell responses were observed at high frequencies (Additional file 1: Figure S1, Additional file 2: Figure S2), suggesting that regulation of these DEGs may be associated with these plant defense responses.

In the case of the *Puccinia* pathosystem, the expression profiles of the wheat DEGs resembled those of their barley orthologues with regard to up- or down-regulation, but often differed with respect to expression kinetics (Fig. 6). Wheat DEGs, that were very early and similarly up-regulated after inoculation with adapted and non-adapted isolates, had barley orthologues showing a delayed response which was more pronounced in the host than in the nonhost interaction (Fig. 6, cluster D). In part, this might be due to the faster development of *Ph* in comparison to *Pt*, as indicated by the microscopy (Additional file 3: Figure S3). Over all clusters, both wheat and barley showed more pronounced gene regulation in response to the *Ph* than to the *Pt* isolate, irrespective of the host or nonhost status of the plant, which indicates again a major effect of the *Puccinia* species per se (Fig. 6). In contrast to *Blumeria* and *Magnaporthe,* photosynthesis-associated genes were over-represented within the clusters of down-regulated genes after *Puccinia* inoculation (Fig. 6, clusters A and C), probably because whole leaf tissue was sampled to study *Puccinia* interactions*,* whereas epidermal tissue was sampled for *Blumeria* and *Magnaporthe*. However, the transcriptional reprogramming after *Puccinia* inoculation also shared obvious commonalities with the *Blumeria* and *Magnaporthe* pathosystems: Within the down-regulated DEGs the functional categories ‘cell wall’ and ‘lipid metabolism’ were significantly over-represented (Fig. 6, clusters A and C), while up-regulated DEGs were often associated with ‘secondary metabolism’, ‘stress’, ‘signaling’, ‘miscellaneous enzymes’, ‘transport’ and ‘amino acid metabolism’ (Fig. 6, clusters D, F and G), in agreement with major *Puccinia*-regulated functional categories determined by RNA sequencing [43].

In summary, a future more detailed analysis of the genes in the most strongly over-represented MapMan bins (with log2 ratio of more than 2 in both species) such as “cell wall” in the *Blumeria* and *Magnaporthe* interactions, “OPP” in the *Puccinia* interaction, and “secondary metabolism” plus “amino acid metabolism” in all pathosystems might guide us towards important genes involved in the plant responses to one specific or several pathosystems.

### Differences in nonhost-specific transcriptional reprogramming between wheat and barley

**Fig. 7:**
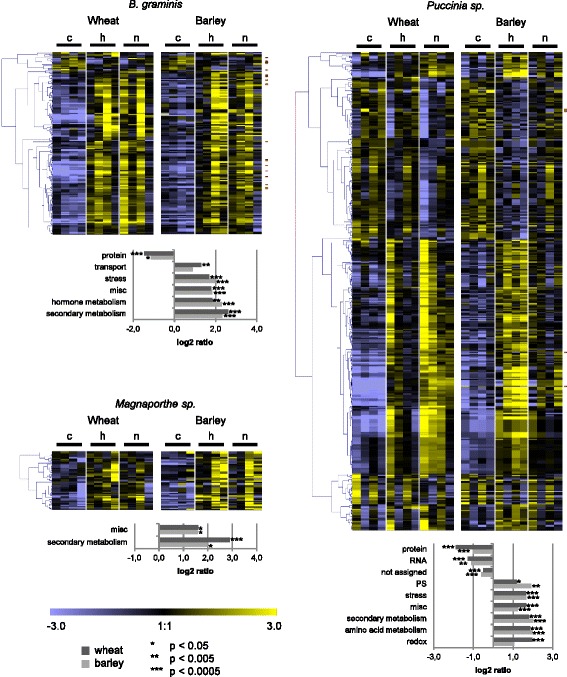
Heat maps of orthologues differentially regulated between host and nonhost interactions in wheat and barley. For *Blumeria, Magnaporthe* and *Puccinia* interactions, wheat probes with differential expression between host and nonhost interaction were filtered for assignment to barley orthologues differentially expressed in the same pathosystem. On the median-centered normalized signal intensities (c: control, h: host interaction, n: nonhost interaction), a hierarchical clustering (Pearson correlation, average linkage) was performed with the software Genesis [88]. The median-centered signal intensities of barley orthologues were sorted accordingly and illustrated in a heat map. Wheat genes that showed significant higher expression in the nonhost compared to the host interaction at one time point, and were assigned to a barley orthologue with a significant higher expression in nonhost compared to host interaction, are marked in red. Over- or underrepresentation of functional MapMan categories and statistical significance according to Fisher Exact Test were calculated with MapMan ORA tool [35, 36]. All probes assigned to orthologues in the other species were taken as reference. The log ratio of presentation in the gene subset and in the reference is shown for BINs found to be significant (BINs comprising 5 or less probes were excluded). PS, photosynthesis; OPP, oxidative pentose phosphate pathway; TCA/org, tricarboxylic acid cycle/organic acid transformation; misc.: miscellaneous

To examine whether wheat and barley share a nonhost-related transcriptional response, in all or any of the pathosystems studied, the nonhost-related DEGs were also searched for matching orthologues (Fig. 3b). Overall, the percentages of DEGs that were assigned an orthologous DEG in the other species were lower than found for the general pathogen-regulated DEGs (Fig. 3b). For the *Blumeria* pathosystem 29% of the orthologue-assigned wheat DEGs and 19% of the orthologue-assigned barley DEGs had *Blumeria-*regulated orthologues in barley and wheat, respectively (Fig. 3b). For the *Magnaporthe* pathosystem the corresponding percentages were 15 and 23%, while for the *Puccinia* pathosystem they were 25 and 32% (Fig. 3b). As for the general pathogen-regulated DEGs, the expression profiles of the nonhost-related wheat DEGs were analyzed by hierarchical clustering, the results being depicted alongside the respective barley orthologues in heat maps (Fig. 7, Additional file 9: Table S4).

With the *Blumeria* pathosystem many wheat DEGs showed higher expression in the nonhost interaction compared to the host interaction at 6 hpi, while this very early response was almost absent in barley (Fig. 7, Additional file 10: Figure S5). Instead, many orthologous barley DEGs showed a higher expression in the nonhost compared to the host interaction at 12 hpi, where most of the respective wheat orthologues were expressed at a lower level in the nonhost interaction, but re-induced at 24 hpi (Fig. 7). A number of DEG orthologues were significantly higher expressed in nonhost compared to corresponding host interactions in both cereal species, but their expression profiles showed different temporal patterns in wheat and barley (Fig. 7, red marks). At the later time points more barley than wheat DEGs showed a specific up-regulation after inoculation with the adapted *Blumeria* isolate (Fig. 7, Additional file 10: Figure S5), suggesting more intense stress of co-option of barley as a susceptible host. Again, it remains open to what extent this reflects a species- or cultivar-specific effect.

With the *Magnaporthe* pathosystem the orthologous nonhost-related DEGs were mostly up-regulated in the later stages of interactions, in particular in the host interactions, this response being more pronounced in barley than in wheat (Fig. 7). Only a few DEGs showed a higher expression in the nonhost than in the respective host interaction, in both wheat and barley, and none of them were shared by the two cereal species.

The regulation of the orthologous nonhost-related DEGs in the *Puccinia* interactions was generally more pronounced after inoculation with *Ph* than after inoculation with *Pt,* in both wheat and barley (Fig. 7), corresponding with the faster development of *Ph* seen in the microscopic study (Additional file 3: Figure S3). Consequently, in wheat most of the nonhost-related DEGs were expressed at higher levels in the nonhost compared to the host interaction, while in barley the opposite was the case (Fig. 7). Only five wheat DEGs and their corresponding three barley orthologues showed a significantly enhanced expression in the nonhost compared to the host interaction, in both wheat and barley (at 36 or 48 hpi, Fig. 7, red marks). The corresponding unigenes (TC407329, TC398344, TC445077, TC410428, TC398384 for wheat, U35_260, U35_16207, U35_16207, U35_16207 and U35_6409 for barley) were annotated as ‘WRKY transcription factor 21’, ‘stress-induced hydrophobic peptide’ and ‘Glucosyl transferase, putative’. However, their expression profiles looked rather different in wheat and barley (Fig. 7).

Taken together these results suggest that there is no significant, common nonhost-related transcriptional response in wheat and barley across pathosystems. To address the possibility that the lack of transcriptional commonalities was a consequence of a too strict orthologue-based approach, an additional, regulon-based approach was used to search for commonalities between wheat and barley nonhost-related DEGs. For each pathosystem all wheat and barley DEGs (Fig. 2b) were pooled and assigned to groups of co-regulated genes by *k*-means and subsequent hierarchical clustering of *k*-means cluster members (Additional file 11: Figure S6). For the pathosystems *Blumeria, Magnaporthe* and *Puccinia* 14, 6 and 8 groups of co-regulated DEGs, which included both wheat and barley DEGs, were identified, respectively, suggesting a conserved co-regulation of several genes in both species (Additional file 11: Figure S6). These groups were subjected to functional MapMan categorization and a BlastN analysis to identify shared gene families within the trans-species *meta*-regulons (Additional file 12: Table S5). However, neither similar functions nor significant sequence similarities were found comparing co-regulated wheat and barley DEGs in each pathosystem (Additional file 12: Table S5). This further supported the conclusion that nonhost-related transcriptional reprogramming in each pathosystem was to a high extent plant-species specific, not being conserved between wheat and barley.

## Discussion

NHR is considered the most common form of disease resistance in plants, protecting all individuals of a plant species against all isolates of a would-be pathogen [10, 44]. Because most studies of NHR have been limited to the comparison of adapted and non-adapted isolates of a single pathosystem, they did not address the broad-spectrum aspect of NHR. In the present study we take a wider view of NHR, comparing multiple pathosystems and two major cereal species of the *Triticeae* tribe of grasses. We asked the question whether similar gene sets are involved in NHR towards different *Triticeae* pathosystems by examining the transcriptional reprogramming in wheat and barley after inoculation with adapted and non-adapted isolates of *Blumeria ff. ssp., Magnaporthe sp.* and *Puccinia sp*. By dissecting the differential response between each host and nonhost interaction from the general pathogen-regulated response in each pathosystem, we aimed to identify those genes that might be involved specifically in NHR. We found a high degree of specificity in the nonhost-related responses between pathosystems, suggesting that different sets of genes are involved in the NHR responses operating against pathogens with different lifestyles. We also found a high degree of specificity in the nonhost-related responses of wheat and barley within a pathosystem, suggesting that independent nonhost barriers were built up against evolving pathosystems after *Triticeae* speciation, and during subsequent host-pathogen co-evolution.

### *Triticeae* activate a general transcriptional regulon in response to different pathogens

With all three pathosystems wheat and barley responded with a significant general pathogen-regulated transcriptional reprogramming that was mostly irrespective of the isolate being adapted or non-adapted. In wheat and barley 13 and 15%, respectively, of the general pathogen-regulated genes were shared by all three pathosystems thus constituting a core pathogenesis-related transcriptome. DEGs in this group, both in wheat and barley, showed a significant over-representation of the functional categories ‘secondary metabolism’, ‘miscellaneous enzymes’, ‘stress’, ‘hormone metabolism’, ‘cell wall’, ‘amino acid metabolism’, ‘lipid metabolism’, ‘signaling’ and ‘transport’ (Additional file 13: Figure S7a, b). A significant portion of the general pathogen-regulated genes (in total 37%) was also shared by at least two different pathosystems, in both wheat and barley, indicating a common plant response to pathogens of different fungal genera. This common response is likely to be triggered by conserved fungal elicitors, PAMPs or effectors, which often trigger similar defense pathways [20, 45, 46].

With the *Blumeria* and *Magnaporthe* pathosystems similar numbers of general pathogen-regulated DEGs were found in wheat and in barley, suggesting a similar quantitative response. In contrast, clearly more genes were differentially regulated in wheat than in barley in response to the *Puccinia* isolates. This would indicate an extraordinary sensitive perception of rust attack by wheat, at least by cv. Renan, which possesses enhanced basal resistance against rust pathogens [47]. The general pathogen-regulated reprogramming can be regarded as the core plant response triggered by fungal PAMPs, as a large part of the general pathogen-regulated DEGs were assigned in the MapMan analysis to protein functions associated with PTI [45]. The majority of the general pathogen-regulated DEGs were up-regulated with the functional categories ‘signaling’ and ‘secondary metabolism’ being significantly over-represented. Many of the ‘signaling’-associated DEGs encoded receptor-like kinases (RLKs), a group of genes associated with pathogen recognition receptors (PRRs) and PTI complexes [48–50]. The majority of the DEGs assigned to ‘secondary metabolism’ were involved in the phenylpropanoid or flavonoid metabolic pathways, which are important for plant defense, by providing precursors for lignification, salicylic acid or phytoalexin production [51]. A common feature of DEGs down-regulated after inoculation with *Blumeria, Magnaporthe* and *Puccinia* isolates was their frequent assignment to ‘cell wall metabolism’. Changes in the plant cell wall’s chemical composition have often been observed following pathogen attack [39, 52]. In bean, cell wall alterations in response to *Colletotrichum* attack were suggested to contribute to plant defense by making the plant cell wall less susceptible to fungal cell wall-lytic enzymes [52–54]. In this study the down-regulation of a number of cellulose-synthesis-associated genes may reflect the necessity to switch from normal to a defense-state of cell-wall metabolism making the cell wall less sensitive to fungal cellulases, which plant pathogens like *Puccinia, Blumeria* and *Magnaporthe* are known to produce [55–57]. Furthermore, the regulation of ‘cell wall’-associated genes may be associated with papillae formation, which in barley have been shown to contain callose, arabinoxylan and cellulose [39].

The sub-sets of general pathogen-regulated genes (in total 63%) that were found only with one of the pathosystems, *Blumeria*, *Magnaporthe* or *Puccinia,* points to plant responses specific for the pathogen genus or species. These may represent part of a PTI response that is associated with the pathogen’s mode of infection and/or pathogen induced changes in the plant that are associated with the specific lifestyle of the pathogen. For example, the formation of *Puccinia* appressoria leads to closure of the invaded stomata [25], which may in turn cause stress-associated metabolic changes in the plant, resulting in transcriptome changes that are not directly linked to plant defense. In addition, the pathosystem-specific DEGs might reflect responses triggered by conserved effectors or, in the case of *Magnaporthe*, also toxins [7, 58]. Such effectors, acting irrespective of the host or nonhost status of the plant, would probably be specific to each pathosystem [20]. The existence of pathosystem-specific effector sets that may act on closely related host and nonhost plants is supported by the description of many effector homologues between *Bgh* and *Bgt* [59].

### Non-adapted pathogens evoke highly specific transcriptional responses in wheat and barley

**Fig. 8:**
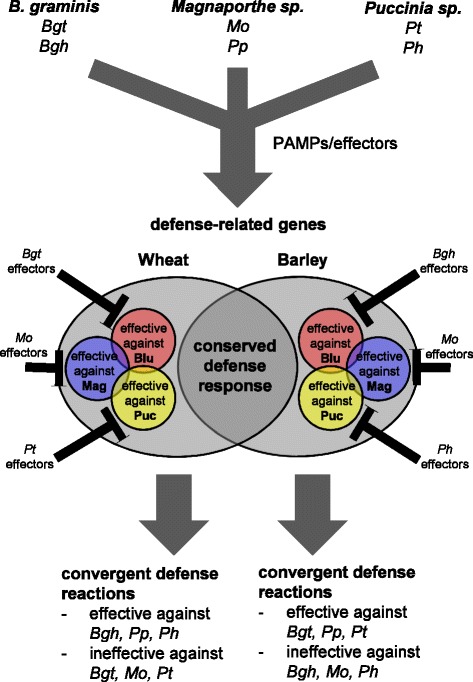
Model of selective suppression of host defense by different pathogens in two *Triticeae* species. Adapted and non-adapted isolates of different pathogen genera evoke a similar transcriptional re-programming in wheat and barley. Different parts of this general response are selectively suppressed by the adapted isolates. This results in a specific differential regulation between host and respective nonhost interactions. Although the outcome of defense reactions against the different pathogens is convergent, also in wheat and barley, they are differently effective against the individual isolates (adapted from [19])

The DEGs significantly differing in their regulation between host and nonhost interactions were identified as a sub-set of the general pathogen-regulated DEGs in a two-step statistical approach. These nonhost specific changes in the transcriptome may be the result of: (i) the ability of the adapted isolate to alter the plant gene transcriptome to create an invasion friendly environment, something the non-adapted isolate is unable to do; (ii) suppression of a part of the general plant defense transcriptome by the adapted isolate to allow successful invasion; and/or (iii) specific defense responses evoked by the non-adapted, but not the adapted isolate. One of the key findings of this work is that, in contrast to the general pathogen-regulated genes, the genes differentially expressed between host and nonhost interactions showed only small overlaps between the three cereal pathosystems. This is in line with findings in barley where transcriptional changes associated with different nonhost interactions were generally specific to the corresponding pair of adapted and non-adapted isolates [19]. The observed pathosystem specificity of nonhost-related responses may in part be explained by the different lifestyles of the pathosystems investigated, each having evolved unique strategies for successful invasion and host immunity suppression. Taken together, our data and those from previous studies [8, 19] propose PAMP-mediated induction of a general defense response, which would be triggered by adapted and non-adapted isolates in all three pathosystems. In a pathosystem specific manner, this general response would then be overlaid by selective suppression, with the non-adapted isolate being unable to sufficiently manipulate the general defense systems of the plant for successful invasion (Fig. 8).

### General pathogen-regulated transcription profiles are conserved in wheat and barley

Having compared the sets of pathogen-responsive genes in wheat and barley to adapted and non-adapted isolates from three distinct pathosystems, we then asked whether the transcriptional profiles had been conserved across these two *Triticeae* species. For this comparison we identified putative orthologues between wheat and barley taking two approaches; (i) an adjusted reciprocal best blast hit (RBH) method and (ii) creation of orthologue clusters using InParanoid [34]. We found that the transcriptional profiles of the orthologous DEGs between wheat and barley for the general pathogen-regulated genes were very similar. Schreiber et al. [60] undertook a comparative transcriptomics study in the *Triticeae*, looking at different tissues and developmental conditions, and also found conservation of expression profiles between wheat and barley. There is also evidence that part of the plant response to fungal pathogens is conserved between monocot and dicot species. Humphry et al. [61] described a regulon of antifungal defense in barley and *Arabidopsis thaliana,* which is co-regulated with known components of the plant defense system (MLO, SNAP34, HvROR2 as homologue of AtPEN1, and HvPDR8 as homologue of AtPEN3). In barley, this highly conserved regulon included 356 ESTs [61], of which, interestingly, 73% were also found in the list of general pathogen-regulated DEGs of barley presented here (Fig. 2a, Additional file 4: Table S1). In conclusion there exists a core pathogenesis-related regulon in wheat and barley for the pathosystems investigated.

### Nonhost-related transcriptional profiles differ between wheat and barley

Besides the fact that only relatively small sets of orthologue-matched nonhost-related DEGs were found, comparison of their transcriptional profiles showed no clear conservation between wheat and barley. The apparent lack of nonhost-related co-regulation of gene orthologues may be the result of (i) wheat and barley using an overlapping set of genes that are differently fine-tuned during defense against different non-adapted isolates, (ii) overlapping gene sets of the general defense response being manipulated differently by the adapted isolates in wheat and in barley, each in a manner that is optimal to the requirements of the respective adapted pathogen (Fig. 8), or (iii) the small sets of orthologue-matched nonhost-specific genes representing false positives in one or both species. Option (ii) may seem most plausible, as during co-evolution with their host plant each adapted isolate would have evolved specific effectors to suppress defense and enable invasion of that specific plant species. Although the effector repertoires of closely related pathogen species such as *Bgt* and *Bgh, Mo* Br116.5 and *Mo* TH6772, or *Pt* and *Ph,* may overlap to a certain extent, pathogen species-specific effectors might be responsible for evoking different transcriptional responses in their host plant. In our study, the wheat transcriptome responded earlier to the adapted *Blumeria* isolate than the barley transcriptome, whereas the response to adapted isolates of *Magnaporthe* and *Puccinia* was weaker in wheat than in barley. These differences in the transcriptional reprogramming were to some extent mirrored in the cytology. In the host interactions *Bgt* evoked an earlier plant cellular response in wheat than *Bgh* in barley, while *Mo* Br116.5 and *Pt* on wheat developed more slowly than *Mo* TH6772 and *Ph* on barley. The differences in fungal development could explain the distinct gene expression profiles of wheat and barley, further emphasizing the impact of pathogen isolate-specific properties. Option (iii), in the case of the *Puccinia* pathosystem, appears unlikely because the orthologous gene set is larger and exhibits an obvious trend for enhanced regulation in a pathogen species- but not nonhost status-dependent manner, i.e. *Ph* induced stronger transcriptional responses than *Pt,* both in wheat and barley (Fig. 7). However, gene orthology does not necessarily predict identical gene function, because neo-functionalization of orthologous genes may occur independently in different plant species [62]. Besides the orthologue mapping, we therefore looked for across species-conserved regulation of genes potentially involved in NHR, by first selecting strictly co-regulated, nonhost-related genes in both wheat and barley, followed by Blast-based analysis of their potential function or gene-family membership (Additional file 11: Figure S6). This revealed *k*-means subclusters in all three pathosystems, which are clearly co-regulated in a nonhost-specific manner across wheat and barley. Some of these, such as subcluster 4 and 9 in the *Blumeria* pathosystem (Additional file 11: Figure S6), showed strong or even qualitative differences between hosts and nonhosts. However, there was no evidence for any functional relatedness of shared family membership of these strictly co-regulated genes. This further substantiated the observation, that NHR in wheat and barley probably relies on different essential responses.

Superimposed upon a common, basal pathogen defense response, wheat and barley may have evolved different responses to a particular pathogen species after the wheat-barley species split which occurred some 10–14 million years ago [60]. These responses might be triggered by stacks of NLR-type resistance genes [20]. While the adapted isolates are constantly overcoming NLR recognition and are efficiently suppressing basal defense responses, other, gradually less adapted isolates would have lost these capabilities and would remain non-adapted isolates. The expected outcome of such a scenario of gradually independent host-pathogen co-evolution would be a considerable non-overlap of nonhost-differentially regulated genes, as described here. Differences in NHR defense responses have also been reported to be host genotype specific in rice and barley. In rice the regulation of defense genes in response to adapted and non-adapted isolates of *Magnaporthe* differed between rice cultivars [63]. A similar genotype-specific response was observed for barley, where NHR to *Pt* in different cultivars depended on a different set of genes [13], indicating that different NHR defense responses have even evolved within plant species.

## Conclusions

With respect to the value of NHR for plant resistance breeding, it may be reasonable to consider the transfer of NHR from one species to the other. In barley, the receptor-like kinase *LEMK1* was shown to confer NHR to *Bgt,* while transient over-expression of *HvLEMK1* was found to reduce the establishment of *Bgt* haustoria in epidermal cells of the wheat cv. Kanzler [64]. Johnston et al. [65] also reported transferring *Ph* resistance from the nonhost species *Hordeum bulbosum* to *H. vulgare*. In wheat, the multi-pathogen resistance gene *Lr34/Yr18/Sr57/Pm38* has been shown to enhance resistance against rusts and powdery mildew when expressed in barley, and against *M. oryzae* in rice [66, 67]. Therefore the global transcriptomic data sets generated within this study provide a valuable resource for further investigation aimed at identifying orthologous NHR as well as non-orthologous host susceptibility components in wheat and barley, that upon transfer across cereal species and mutagenesis, respectively, may confer durable host resistance.

## Methods

### Cultivation of plants and pathogens and inoculation procedures

Pathogen inoculation experiments were performed on barley cultivar Vada and on wheat cultivar Renan.

For *Blumeria* inoculation plants were grown in plastic pots (14 cm diameter) with standard compost soil mixture (from IPK, Gatersleben greenhouse nursery) without fertilization. Seedlings were allowed to grow in a plant climate chamber (NEMA Industrietechnik GmbH) at 19 °C with 65% relative humidity (RH) during night and 23 °C with 50% RH during day with 16 h of photoperiod. *Bgh* strain CH4.8 and *Bgt* Swiss field isolate FAL92315 were used to inoculate the plants with a spore density of 50–80 conidia mm^−2^ by shaking the spores over the test plants in a settling tower of approximately 60 × 60 × 60 cm. Inoculated and non-inoculated control plants were incubated in the same plant climatized room mentioned above with indirect sunlight until epidermal peeling.

The *Magnaporthe* isolates CD180 from *Pennisetum* sp., TH6772 from rice and Br116.5 from wheat were kindly provided by Didier Tharreau (CIRAD Montpellier, France), by the institute of Biochemistry, Tamagawa University (Machida-shi, Tokyo, Japan) and by Yukio Tosa (Kobe University, Japan [68]), respectively. Cultivation of plants and fungi as well as inoculum preparation were performed as described before [69]. The conidia suspension was adjusted to 400,000 spores ml^−1^, diluted 1:2 with surfactant (2 g l^−1^ gelatin, 1 ml l^−1^ Tween) to a final concentration of 200,000 conidia ml^−1^ and spray-inoculated onto plants. For mock-inoculation plants were sprayed with 50% surfactant solution. During the first 24 h after inoculation, plants were incubated at 24 °C and 100% relative humidity in the dark. Afterwards, they were cultivated under a plastic hood with growing conditions. Preliminary inoculation tests were performed to find adequate isolates for host and nonhost interactions with barley and wheat. Isolate CD180 was confirmed to be non-adapted on different wheat and barley cultivars (unpublished data, [30]). Isolate Br116.5 evoked distinct blast lesions on wheat cv. Renan (Fig. 1a), but extraordinary severe blast symptoms and leaf death on barley. Instead TH6772, nonpathogenic on wheat cv. Renan, evoked a similar disease phenotype of distinct blast lesions on barley cv. Vada (Fig. 1a).

For rust inoculation, fifty seedlings of the barley cultivar Vada and the wheat cultivar Renan were grown in boxes (37 × 39 cm). Ten days after sowing, the first leaves were fixed in horizontal position, the adaxial side facing up. *Puccinia hordei,* 1.2.1 isolate, and *Puccinia triticina*, BRW96258 isolate, were used for inoculation. For mock inoculation only Lycopodium powder was used. Inoculations were performed with 10 mg of rust urediniospores per box in a settling tower [70]. The inoculated seedlings were incubated overnight in a dew chamber for 10 h (17–18 °C) at 100% relative humidity and darkness and then transferred to a greenhouse compartment.

### Cytological investigations

To study the microscopic response to *Bgh* and *Bgt*, one-week-old primary leaves were inoculated with 8–10 conidia mm^−2^ at whole plant level. After inoculation, pots were incubated in the plant climatized room mentioned above until the respective harvesting time point. For visualization of the fungus, leaves were stained with Coomassie solution (0.3% Coomassie R250 stain, 7.5% Trichloroacetic acid (TCA), 50% methanol) for 10–15 min, washed and stored in water.

Barley and wheat leaves inoculated with *Magnaporthe* isolates were prepared for microscopic investigation of early fungal developmental stages as described previously [69, 71]. Harvested leaves were placed on Whatman paper soaked with 25% acetic acid in ethanol (*v*/v). When cleared of chlorophyll, leaves were microscopically examined in water with a DMBRE microscope (Leica Microsystems, Wetzlar, Germany).

For histological analysis of host and nonhost interactions of barley and wheat with *Puccinia*, two leaf segments, 2–3 cm long, were excised from the inoculated seedlings at three time points, 12, 24 and 36 hpi. The leaf segments were fixed and cleared by boiling for 1.5 min in lactophenol/ethanol (1:2 v/v) in a boiling water bath, and left overnight in this mixture at room temperature. Leaf segments were stained in 0.1% Uvitex 2B (Ciba-Geigy, Switzerland) as described by Hoogkamp et al. [72]. The preparations were examined with an epi-fluorescence microscope Axiophot (Zeiss, Germany).

### Microarray hybridization and data analysis

For each pathosystem (*Blumeria, Magnaporthe* or *Puccinia*) wheat and barley leaf material was harvested at four different time points after inoculation with an adapted or non-adapted isolate or the respective mock-treatment in three independent experiments (giving 3 times 12 samples per plant-pathosystem combination). For *Blumeria* and *Magnaporthe* interactions, the abaxial epidermis of 20–200 inoculated primary leaves was sampled, while in case of the *Puccinia* interactions whole leaf material of 40 plants was harvested. RNA from all samples was extracted using the RNeasy Plant Mini Kit (Qiagen, Hilden, Germany). DNA was eliminated from RNA samples by on-column digestion with the RNase-Free DNase Set (Qiagen). In the case of *Puccinia* treated samples, the Ambion® TURBO DNA-free™ DNase Kit was used for DNA elimination. After passing quality control (using Agilent 2100 Bioanalyzer, Agilent Technologies, Inc.) RNA was hybridized to Agilent 44 k oligonucleotide arrays using One Color Microarray-Based Gene Expression Analysis- Low Input Quick Amp Labeling, v 6.5, as recommended (Agilent Technologies). The Agilent wheat Expression Microarray (design ID 22297, Agilent technologies, Santa Clara, USA) and a custom barley microarray SCRI_Hv35_44k_v1 (Agilent design ID 20599) [73–75] were used. Raw microarray data were deposited at ArrayExpress [76, 77] (www.ebi.ac.uk/arrayexpress, accession numbers: E-MTAB-2916 for barley-*Blumeria*, E-MTAB-3803 for wheat-*Blumeria*, E-MTAB-5634 for barley-*Magnaporthe*, E-MTAB-5635 for wheat-*Magnaporthe*, E-MTAB-5655 for barley-*Puccinia*, E-MTAB-5656 for wheat-*Puccinia*).

Microarray data analyses were performed on each plant-pathogen combination separately but following the same workflow. Raw microarray data were background-corrected and quantile normalized using GeneSpring GX software (Agilent Technologies). To identify possible outlier samples, principal component analysis and clustering of the data was performed. In the case of the barley-*Puccinia* interaction, this revealed that samples of one biological replicate did not cluster with the other two replicates (Additional file 14: Figure S8). Therefore, the samples of this single experiment were excluded from further data analyses. For statistical analysis, the mean signal intensities of each time-treatment combination were calculated. Subsequently, data were filtered for probes flagged ‘detected’ in at least one of the 12 time-treatment combinations. On the filtered data, a combination of three statistical approaches were applied to identify probes with significant regulation in host or nonhost interactions compared to control treatment. The first approach was a paired t-test on the differences between host vs. control and nonhost vs. control taking the averages across the investigated time points (‘static’ approach). Probes that passed the statistical criteria (fold change ≥2 or ≤ − 2, α ≤ 0.05 after Benjamini-Hochberg-correction) were summarized to a non-redundant list. In parallel, in a second and third approach ‘single-time point’ analyses were performed using One Way ANOVA with post hoc Tukey HSD or an unpaired t-test, respectively. In each approach probes significantly regulated in host vs. control or nonhost vs. control (fold change ≥2 or ≤ −2, α ≤ 0.05) at the different time points were pooled. Only the probes that passed both single-time point approaches were added to the list resulting from the first, static approach to give a non-redundant list of ‘general pathogen-regulated probes’. To identify probes with a significantly different regulation in host compared to nonhost interaction, a One Way ANOVA with post hoc Tukey HSD was applied to the list of general pathogen-regulated probes. Probes showing significant differential expression in host and nonhost interaction (fold change ≥2 or ≤ −2, α ≤ 0.05) for at least one time point investigated were summarized to a non-redundant list of ‘nonhost-related differentially regulated’ probes. For each plant-pathogen interaction the lists of probes found to be ‘general pathogen-regulated’ and ‘differentially regulated between host and nonhost interaction’ are given in Additional file 4: Table S1 and Additional file 6: Table S2, respectively (according to Venn diagrams in Fig. 2). The reliability of the array-derived transcriptional data were verified by RT-qPCR with primer pairs for a number of (randomly chosen) *Magnaporthe*-regulated transcripts and revealed good agreement between results from both methods (Additional file 15: Figure S9). Primer used in this analysis listed in Additional file 16: Table S6.

### Meta analyses of transcriptome data

Probes of the wheat and the barley microarrays were assigned to unigenes of the DFCI *Triticum aestivum* Gene Index (TaGI) database [78, 79] or the HarvEST database (HarvEST:Barley, v. 1.83) [80], respectively. Sequences were re-annotated using Basic Local Alignment Tool (BLAST) [81, 82] with the databases of the National Center for Biotechnology Information (NCBI) [83] and the Blast2Go software [84]. The oligonucleotide sequences of the barley microarray were also assigned to the contigs of the barley genome published by the International Barley Genome Sequencing Consortium (IBSC) [85]. However, many oligonucleotides were assigned redundantly to single cds sequences of IBSC, so that a transcriptome analysis based on these cds sequences would have required a previous data reduction. To circumvent the loss of possibly meaningful data, the barley transcriptome data were analyzed based on the unigene sequences to which the microarray was originally designed [73–75]. Unigene sequences present on the barley and the wheat microarray were assigned to functional ‘MapMan’ categories using Mercator pipeline [86, 87]. Over-representation analyses (ORA) of functional categories in lists of regulated unigenes were performed with the web-based MapMan ORA tool [35, 36]. For orthologue identification between wheat and barley unigenes, a reciprocal best hit (RBH) analysis between the TaGI and the HarvEST unigenes was performed. Additionally, orthologue clusters of the TaGI wheat unigenes and the barley cds sequences of IBSC (2012) were determined using InParanoid [34]. The results of both approaches were combined to a matching table linking 57.8% of the wheat to 38.0% of the barley probes (Additional file 7: Table S3). In order to test whether the overlap between wheat and barley general pathogen-regulated DEGs was larger than by chance, (i) the same number of wheat (barley) probes that were significant were sampled translated into their respective orthologues and compared with the barley (wheat) list of significant genes. Alternatively (ii), the wheat (barley) probes declared significant that had an orthology in barley (wheat) were counted and the same number of wheat (barley) genes featuring an orthologue was sampled translated into their orthologues and compared to the barley (wheat) list of genes (Additional file 8: Data S1). In each case, these analyses revealed the numbers of matched orthologues within random gene sets to be lower than within the identified DEGs. Hierarchical and *k*-means clusterings were performed using the software Genesis (version 1.8) [88].

## Additional files

Additional file 1: Figure S1. Quantitative cytology of wheat and barley interactions with *Blumeria* isolates. Wheat cv. Renan and barley cv. Vada were inoculated with *Blumeria graminis* f. sp*. tritici (Bgt)* and *Blumeria graminis* f. sp*. hordei (Bgh).* At timepoints indicated interaction sites with fungal appressoria were cytologically evaluated for presence of elongating secondary hyphae (ESH). In addition, the autofluorescence response of the plant was evaluated and assigned to categories indicated. Columns represent mean category percentages of 100 interactions sites counted from four leaves in two biological experiments. (PDF 58 kb)

Additional file 2: Figure S2. Quantitative cytology of wheat and barley interactions with *Magnaporthe* isolates. Wheat cv. Renan and barley cv. Vada were inoculated with *Magnaporthe oryzae (*isolate Br116.5 or TH6772, respectively) and *Pyricularia penniseticola* (isolate CD180)*.* At timepoints indicated interaction sites with fungal conidiospores were cytologically evaluated for presence of germ tube, appressorium and fungal growth inside the attacked epidermal cell. In addition, at 24 and 48 h after inoculation the autofluorescence response of the plant was evaluated and assigned to categories indicated. Columns represent mean category percentages of at least 100 interactions sites counted from two to four leaves in three (wheat) and two (barley) biological experiments. (PDF 88 kb)

Additional file 3: Figure S3. Quantitative cytology of wheat and barley interactions with *Puccinia* isolates. Wheat cv. Renan and barley cv. Vada were inoculated with *Puccinia triticina* and *Puccinia hordei.* At 12, 24 and 36 h past inoculation (hpi) interaction sites with fungal appressoria were cytologically evaluated for the developmental stage of the fungus (appressorium, substomatal vesicle, infection hyphae and haustorial mother cell). At 48 hpi infection units that had formed a haustorial mother cell were assigned for categories of further development as indicated. Columns represent category percentages of approx. 100 interactions sites of two investigated leaves. (PDF 74 kb)

Additional file 4: Table S1. Lists of wheat and barley genes general regulated after pathogen inoculation (lists of the subsets according to Venn diagrams in Fig. 2a). Signal values are an output of the GeneSpring GX software and given as log base 2 values. Fold changes are calculated accordingly. (XLSX 22727 kb)

Additional file 5: Figure S4. Numbers of DEGs found to be up- or down-regulated in host or nonhost interactions compared to mock inoculations (according to average log fold changes of host vs. control and nonhost vs. control at different time points). (PDF 24 kb)

Additional file 6: Table S2. Lists of wheat and barley genes differentially regulated in host and nonhost interactions with *Blumeria, Magnaporthe* and *Puccinia* (lists of the subsets according to Venn diagrams in Fig. 2b). Signal values are an output of the GeneSpring GX software and given as log base 2 values. Fold changes are calculated accordingly. (XLSX 3027 kb)

Additional file 7: Table S3. Linking of wheat and barley orthologue candidates. (XLSX 2951 kb)

Additional file 8: Data S1. Simulations of orthologue assignment within random wheat (barley) gene sets to general pathogen regulated barley (wheat) DEGs showing that the overlap found between wheat and barley general pathogen-regulated DEGs was larger than by chance. (PDF 10 kb)

Additional file 9: Table S4. IDs, median-centered intensity values and Blast annotation of orthologue-matched DEGs represented in the heat maps of Figs. 4, 5, 6 and 7. (XLSX 1559 kb)

Additional file 10: Figure S5. Numbers of genes found to be differentially regulated between host and nonhost interactions at different time points in wheat or barley after inoculation with adapted and non-adapted isolates of *Blumeria*, *Magnaporthe* and *Puccinia* (according to statistical analysis of microarray data). (PDF 51 kb)

Additional file 11: Figure S6. Results of *k*-means clustering followed by hierarchical clustering on the nonhost-related DEGs of wheat and barley in each pathosystem. In an independent approach to identify co-regulated orthologues, a *k*-means clustering followed by a hierarchical clustering of individual *k*-means cluster members was carried out for both cereal species and respective interactions. For this, the normalized signal intensity values of host and nonhost DEGs (Fig. 2b) of the respective pathosystem were median-centered separately for wheat and barley in Genesis software (release 1.8.0, [88]). The median-centered signal values of both species were pooled for each pathosystem to form a single list, and a *k*-means clustering analysis was done in Genesis with default settings. Individual *k*-means clusters were then hierarchical clustered in Genesis with Pearson correlation, average linkage settings. To identify co-regulated regulons, sub-clusters with the occurrence of both wheat and barley probes (left clustering image; nodes with sub-clusters highlighted in color) were manually identified and extracted. A zoomed in image of these sub-clusters is shown on the right. Please note the occurrence of both wheat (A_99_) and barley (CUST_) probe IDs in the same node. In order to determine the gene function relation among these co-regulated wheat and barley DEGs, MapMan (3.5.1R2) and BlastN analyses (default parameters, BLASTN 2.2.29+) were carried out. (PDF 3400 kb)

Additional file 12: Table S5. IDs, median-centered intensity values and MapMan annotation of *k*-means and hierarchical clustering subclusters (Additional file 11: Figure S6), and results of BlastN analysis within the subclusters. (XLSX 12681 kb)

Additional file 13: Figure S7. Overrepresentation analysis of functional MapMan categories within the intersections of general pathogen regulated wheat and barley DEGs (a) and DEGs with differential expression between host and nonhost interaction (b) found for the three pathosystems *Blumeria, Magnaporthe* and *Puccinia*. Over- or underrepresentation and statistical significance according to Fisher Exact Test were calculated with MapMan ORA tool [35, 36]. All probes assigned to MapMan BINs were taken as reference. The log ratio of presentation in the gene set and in the reference is shown for BINs found to be significant (BINs comprising 5 or less probes were excluded). (PDF 14 kb)

Additional file 14: Figure S8. Clusterings of *Puccinia* microarray data leading to the decision to exclude one of three biological barley replicates. Ideally, the replications (1, 2, 3) from the same treatment (mock, host, nonhost) and time point (12, 24, 36 and 48 hpi) should cluster together. The clustering of chips was very clear in case of wheat*/Puccinia* interaction (**a**). In case of barley/*Puccinia* this was not the case: 7 out of 12 chips from replication 1 did not cluster into their respective subclades (**b**) (coloured arrows indicate chips out of their respective clusters). Therefore the complete replication 1 of barley/*Puccinia* interactions was omitted from the final analysis, resulting in a great enrichment of host/nonhost differentially regulated genes (11 according to analysis with all 3 replicates, 1824 according to analysis without replicate 1). (PDF 1063 kb)

Additional file 15: Figure S9. Validation of microarray gene expression profiles by quantitative reverse transcription PCR (qRT-PCR). For selected candidate genes in the barley-*Magnaporthe* pathosystem, normalized signal intensities resulting from the microarray analyses (data points show averages and standard errors of three biological replicates) were compared with relative transcript abundancies resulting from qRT-PCR analysis of an independent experiment (bars show averages and standard deviations of two technical replicates). qRT-PCR was performed as described [69]. Asterisks indicate time points for which microarray analyses showed a significant differential expression during nonhost interaction compared to host interaction. In x-marked samples the respective candidate gene transcript was not detectable by qRT-PCR. (PDF 152 kb)

Additional file 16: Table S6. Primer combinations used for validation of microarray gene expression profiles by qRT- PCR (Additional file 15: Figure S9). (PDF 21 kb)

## Abbreviations

*Bgh*: *Blumeria graminis* f. sp. *hordei*
*Bgt*: *Blumeria graminis* f. sp. *tritici*
BLAST: Basic local alignment tool
DEG: Differentially expressed gene
ETI: Effector-triggered immunity
HMC: Haustorial mother cell
hpi: Hours past inoculation
HR: Hypersensitive response
*Mo*: *Magnaporthe oryzae*
NHR: Nonhost resistance
ORA: Over-representation analysis
PAMP: Pathogen-associated molecular pattern
*Ph*: *Puccinia hordei*
*Pp*: *Pyricularia penniseticola*
PRR: Pathogen recognition receptor
*Pt*: *Puccinia triticina*
PTI: PAMP-triggered immunity
RBH: Reciprocal best hit
RH: Relative humidity
RLK: Receptor-like kinase
