# Supplementary material for: A comparative analysis of nonhost resistance across the two Triticeae crop species wheat and barley
Source: BMC Plant Biol. 2017 Dec 4;17:232. doi: 10.1186/s12870-017-1178-0 (PMC5715502; doi:10.1186/s12870-017-1178-0)
Supplement: Supplementary file 1 — Quantitative cytology of wheat and barley interactions with Blumeria isolates. Wheat cv. Renan and barley cv. Vada were inoculated with Blumeria graminis f. sp. tritici (Bgt) and Blumeria graminis f. sp. hordei (Bgh). At timepoints indicated interaction sites with fungal appressoria were cytologically evaluated for presence of elongating secondary hyphae (ESH). In addition, the autofluorescence response of the plant was evaluated and assigned to categories indicated. Columns represent mean category percentages of 100 interactions sites counted from four leaves in two biological experiments. (PDF 58 kb) [file 12870_2017_1178_MOESM1_ESM.pdf]

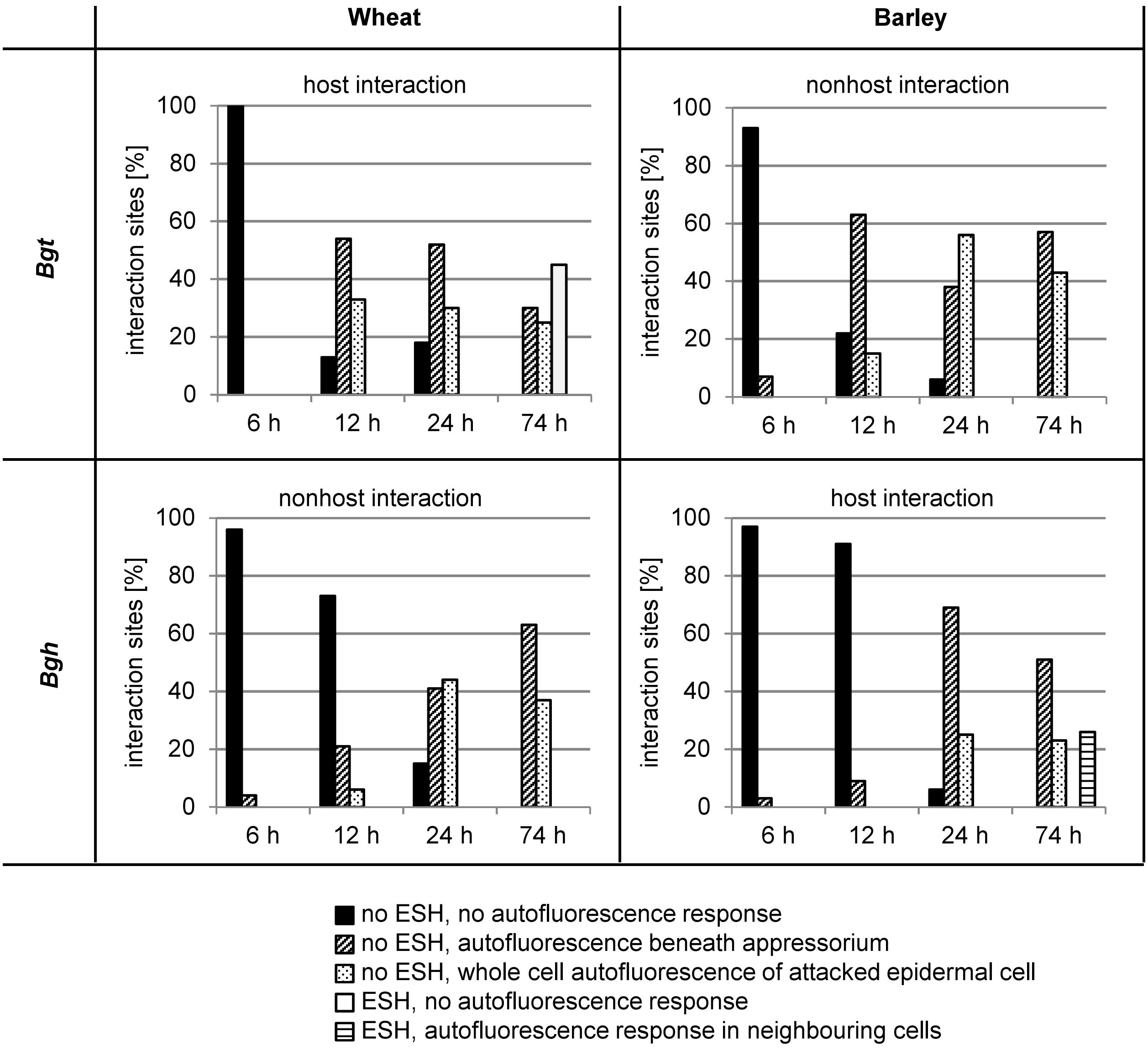


**Figure S1.** Quantitative cytology of wheat and barley interactions with *Blumeria* isolates. Wheat cv. Renan and barley cv. Vada were inoculated with *Blumeria graminis* f. sp. *tritici (Bgt)* and *Blumeria graminis* f. sp. *hordei (Bgh).* At timepoints indicated interaction sites with fungal appressoria were cytologically evaluated for presence of elongating secondary hyphae (ESH). In addition, the autofluorescence response of the plant was evaluated and assigned to categories indicated. Columns represent mean category percentages of 100 interactions sites counted from four leaves in two biological experiments.
