# Supplementary material for: A comparative analysis of nonhost resistance across the two Triticeae crop species wheat and barley
Source: BMC Plant Biol. 2017 Dec 4;17:232. doi: 10.1186/s12870-017-1178-0 (PMC5715502; doi:10.1186/s12870-017-1178-0)
Supplement: Supplementary file 2 — Quantitative cytology of wheat and barley interactions with Magnaporthe isolates. Wheat cv. Renan and barley cv. Vada were inoculated with Magnaporthe oryzae (isolate Br116.5 or TH6772, respectively) and Pyricularia penniseticola (isolate CD180). At timepoints indicated interaction sites with fungal conidiospores were cytologically evaluated for presence of germ tube, appressorium and fungal growth inside the attacked epidermal cell. In addition, at 24 and 48 h after inoculation the autofluorescence response of the plant was evaluated and assigned to categories indicated. Columns represent mean category percentages of at least 100 interactions sites counted from two to four leaves in three (wheat) and two (barley) biological experiments. (PDF 88 kb) [file 12870_2017_1178_MOESM2_ESM.pdf]

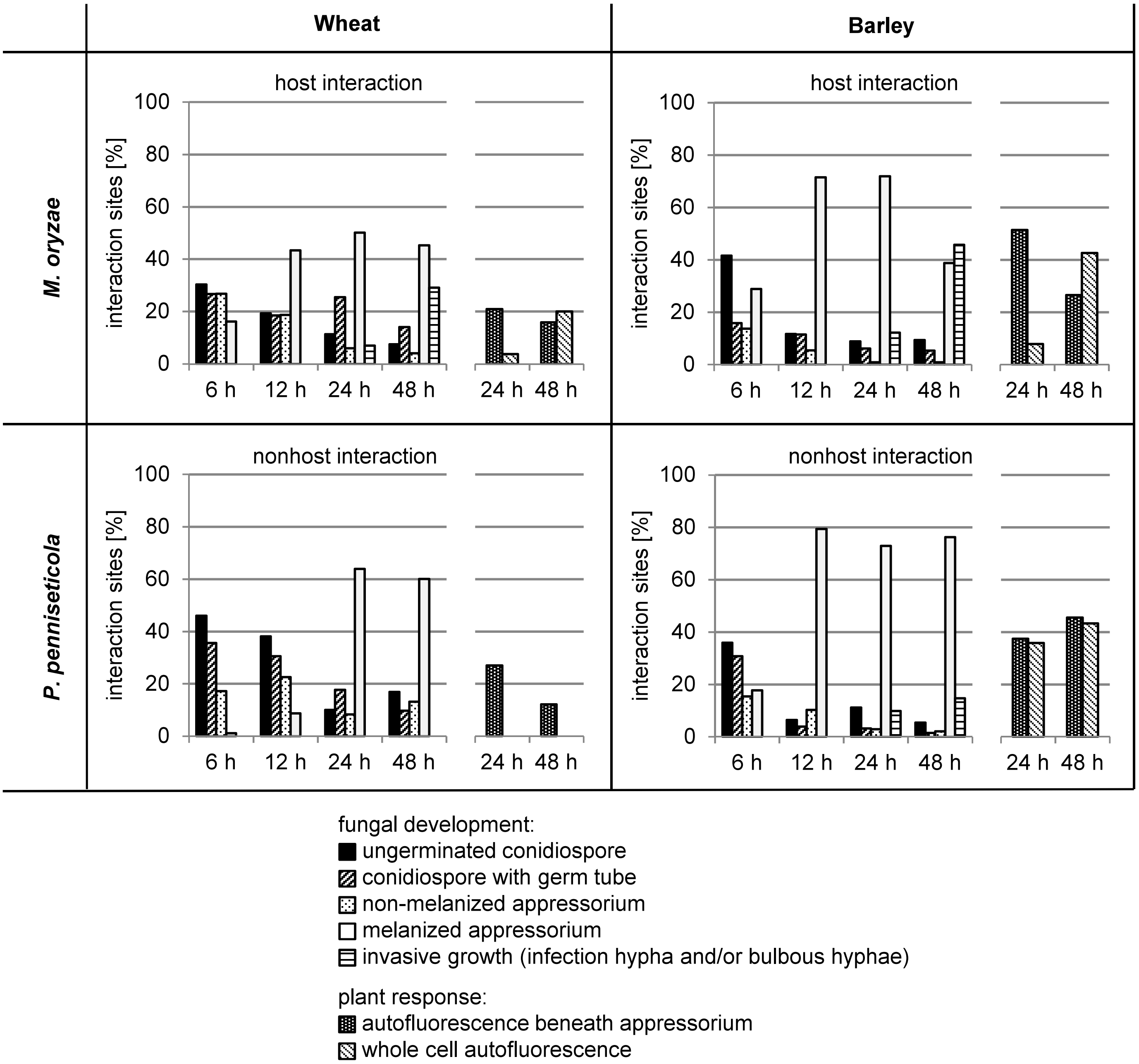

Figure S2. Quantitative cytology of wheat and barley interactions with *Magnaporthe* isolates. Wheat cv. Renan and barley cv. Vada were inoculated with *Magnaporthe oryzae (*isolate Br116.5 or TH6772, respectively) and *Pyricularia penniseticola* (isolate CD180). At timepoints indicated interaction sites with fungal conidiospores were cytologically evaluated for presence of germ tube, appressorium and fungal growth inside the attacked epidermal cell. In addition, at 24 and 48 hours after inoculation the autofluorescence response of the plant was evaluated and assigned to categories indicated. Columns represent mean category percentages of at least 100 interactions sites counted from two to four leaves in three (wheat) and two (barley) biological experiments.
