# Supplementary material for: A comparative analysis of nonhost resistance across the two Triticeae crop species wheat and barley
Source: BMC Plant Biol. 2017 Dec 4;17:232. doi: 10.1186/s12870-017-1178-0 (PMC5715502; doi:10.1186/s12870-017-1178-0)
Supplement: Supplementary file 3 — Quantitative cytology of wheat and barley interactions with Puccinia isolates. Wheat cv. Renan and barley cv. Vada were inoculated with Puccinia triticina and Puccinia hordei. At 12, 24 and 36 h past inoculation (hpi) interaction sites with fungal appressoria were cytologically evaluated for the developmental stage of the fungus (appressorium, substomatal vesicle, infection hyphae and haustorial mother cell). At 48 hpi infection units that had formed a haustorial mother cell were assigned for categories of further development as indicated. Columns represent category percentages of approx. 100 interactions sites of two investigated leaves. (PDF 74 kb) [file 12870_2017_1178_MOESM3_ESM.pdf]

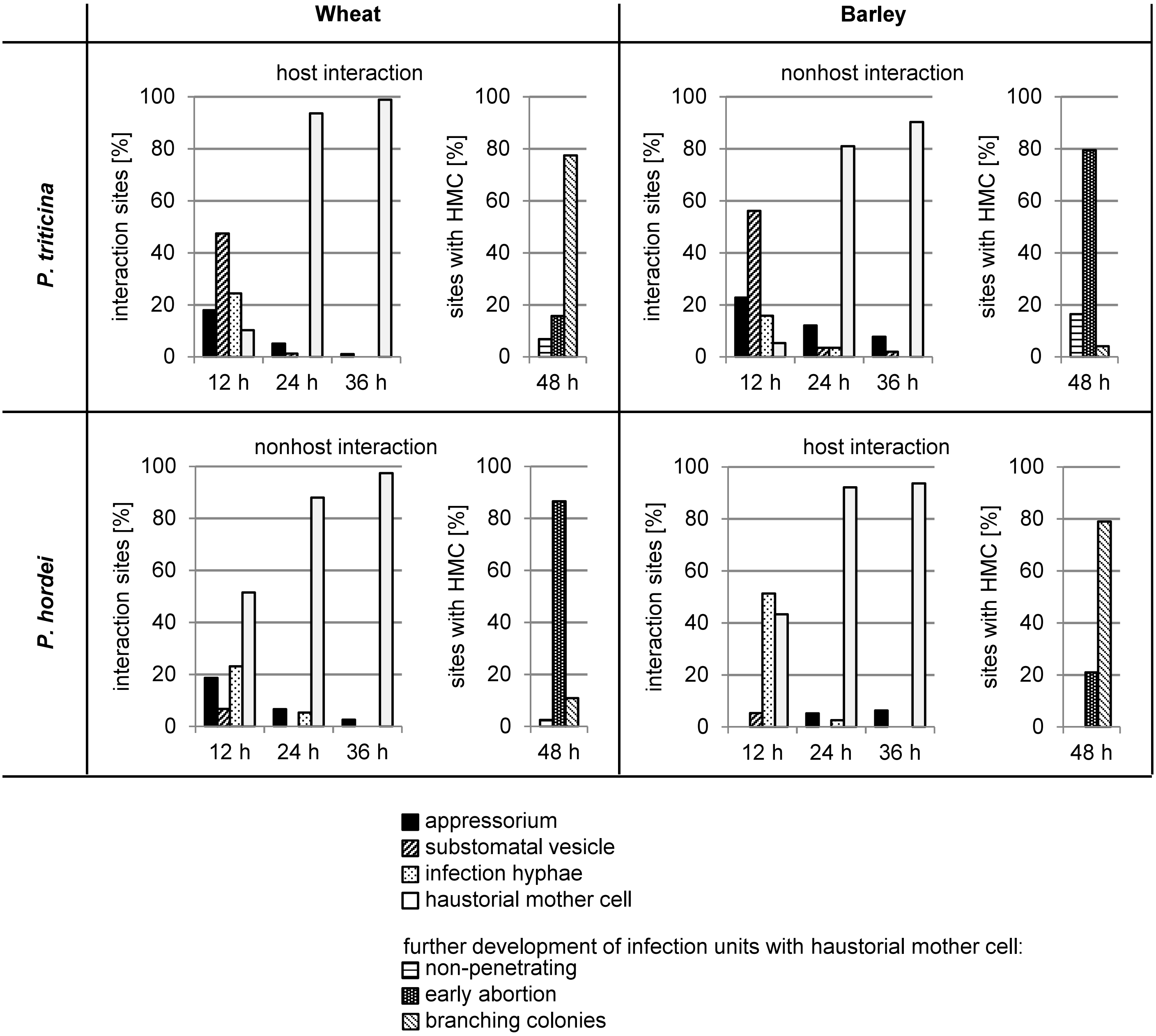

Figure S3. Quantitative cytology of wheat and barley interactions with *Puccinia* isolates. Wheat cv. Renan and barley cv. Vada were inoculated with *Puccinia triticina* and *Puccinia hordei.* At 12, 24 and 36 hours past inoculation (hpi) interaction sites with fungal appressoria were cytologically evaluated for the developmental stage of the fungus (appressorium, substomatal vesicle, infection hyphae and haustorial mother cell). At 48 hpi infection units that had formed a haustorial mother cell were assigned for categories of further development as indicated. Columns represent category percentages of approx. 100 interactions sites of two investigated leaves.
