# Supplementary material for: A comparative analysis of nonhost resistance across the two Triticeae crop species wheat and barley
Source: BMC Plant Biol. 2017 Dec 4;17:232. doi: 10.1186/s12870-017-1178-0 (PMC5715502; doi:10.1186/s12870-017-1178-0)
Supplement: Supplementary file 5 — Numbers of DEGs found to be up- or down-regulated in host or nonhost interactions compared to mock inoculations (according to average log fold changes of host vs. control and nonhost vs. control at different time points). (PDF 24 kb) [file 12870_2017_1178_MOESM5_ESM.pdf]

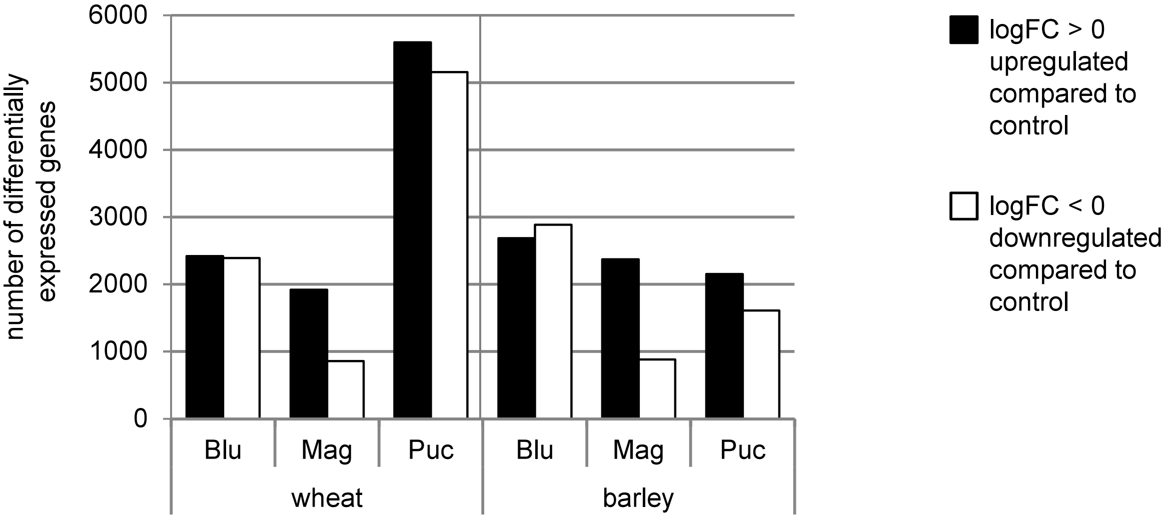

Figure S4. Numbers of DEGs found to be up- or down-regulated in host or nonhost interactions compared to mock inoculations (according to average log fold changes of host vs. control and nonhost vs. control at different time points).
