# Supplementary material for: A comparative analysis of nonhost resistance across the two Triticeae crop species wheat and barley
Source: BMC Plant Biol. 2017 Dec 4;17:232. doi: 10.1186/s12870-017-1178-0 (PMC5715502; doi:10.1186/s12870-017-1178-0)

# Magnaporthe

```
Magnaporthe

Probes on Barleychip: 42348
Probes on Wheatchip: 43635
Barleyprobes in Ortholist: 16079
Wheatprobes in Ortholist: 25224
DE barley: 3252
DE wheat: 2777
DE barley that are in the Ortholist 1380
DE wheat: 2777
DE wheat that are in the Ortholist 1763
_1_ wheat DE genes that have a barley ortholog, barley genes counted: 1473
_2_ Fraction of orthogenes vs all, wheat counted: 0.57806806462702
_3_ Fraction of barleyDE in barleyortholist: 0.0858262329746875
Expected overlap based on _1_ * _3_: 126.422041171715
wheat DE genes that have a DE barley ortholog, barley genes counted: 533
_1_ barly DE genes that have a wheat ortholog, wheat genes counted: 2387
_2_ Fraction of orthogenes vs all, barley counted: 0.379687352413337
_3_ Fraction of wheatDE in wheatortholist: 0.0698937519822391
Expected overlap based on _1_ * _3_: 166.836385981605
DE barley that have a DE wheat ortholog, wheat genes counted: 731
```

**Wheat experiment simulation**
**wheat probes selected from chip 2777**

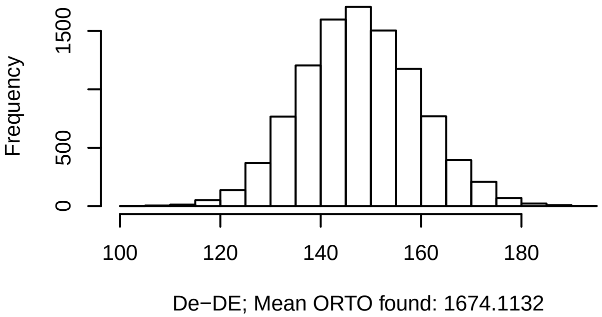


**Barley experiment simulation**
**barley probes selected from chip 3252**

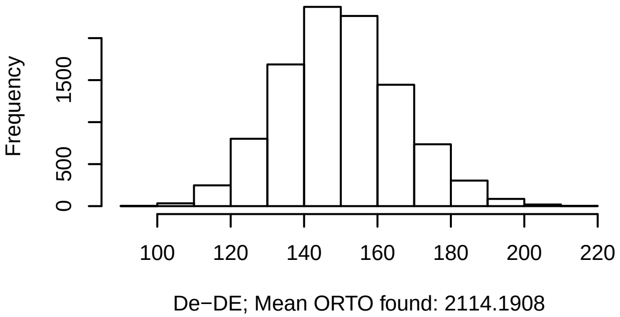


**Wheat experiment simulation**
**barley! uniq. probes w. ortholog selected 1473**

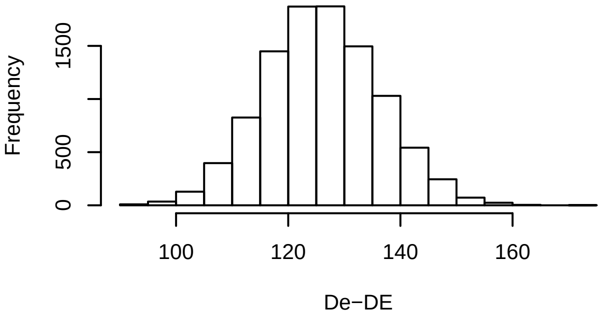


**Barley experiment simulation**
**wheat! uniq. probes w. ortholog selected 2387**

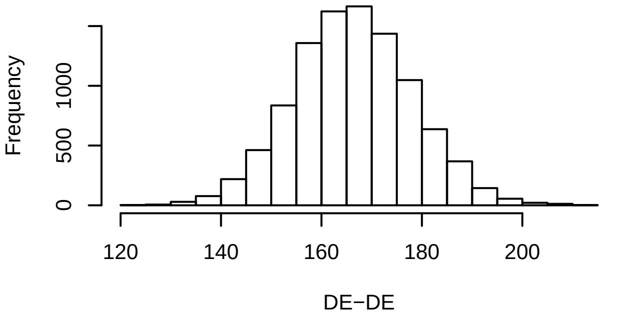

# Blumeria

```
Blumeria

Probes on Barleychip: 42348
Probes on Wheatchip: 43635
Barleyprobes in Ortholist: 16079
Wheatprobes in Ortholist: 25224
DE barley: 5570
DE wheat: 4811
DE barley that are in the Ortholist 2322
DE wheat: 4811
DE wheat that are in the Ortholist 3152
_1_ wheat DE genes that have a barley ortholog, barley genes counted: 2636
_2_ Fraction of orthogenes vs all, wheat counted: 0.57806806462702
_3_ Fraction of barleyDE in barleyortholist: 0.144411965918279
Expected overlap based on _1_ * _3_: 380.669942160582
wheat DE genes that have a DE barley ortholog, barley genes counted: 946
_1_ barly DE genes that have a wheat ortholog, wheat genes counted: 3807
_2_ Fraction of orthogenes vs all, barley counted: 0.379687352413337
_3_ Fraction of wheatDE in wheatortholist: 0.124960355217253
Expected overlap based on _1_ * _3_: 475.724072312084
DE barley that have a DE wheat ortholog, wheat genes counted: 1306
```

**Wheat experiment simulation**
**wheat probes selected from chip 4811**

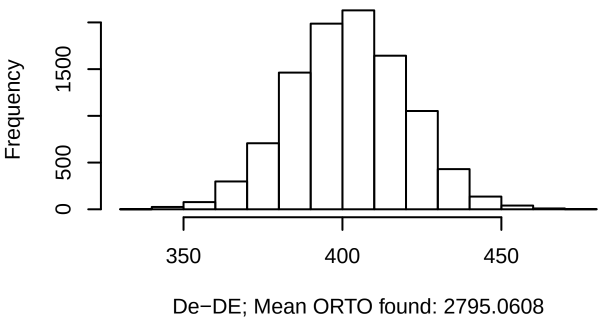


**Barley experiment simulation**
**barley probes selected from chip 5570**

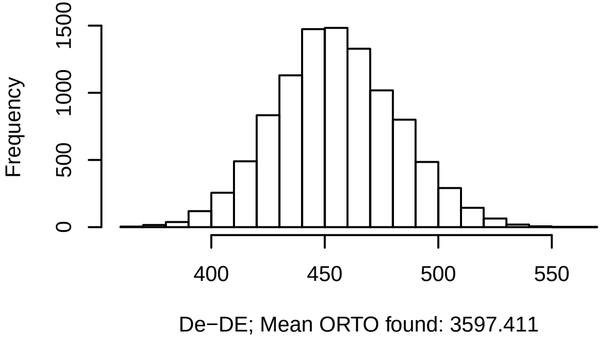


**Wheat experiment simulation**
**barley! uniq. probes w. ortholog selected 2636**

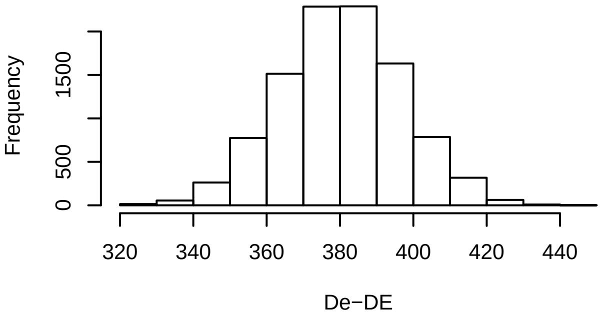


**Barley experiment simulation**
**wheat! uniq. probes w. ortholog selected 3807**

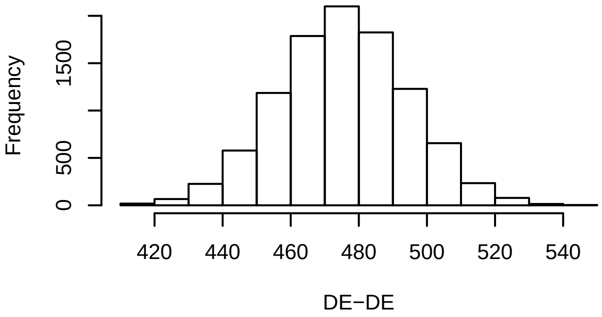

# Puccinia

Puccinia

Probes on Barleychip: 42348
Probes on Wheatchip: 43635
Barleyprobes in Ortholist: 16079
Wheatprobes in Ortholist: 25224
DE barley: 3763
DE wheat: 10756
DE barley that are in the Ortholist 1630
DE wheat: 10756
DE wheat that are in the Ortholist 7169
_1_ wheat DE genes that have a barley ortholog, barley genes counted: 5501
_2_ Fraction of orthogenes vs all, wheat counted: 0.57806806462702
_3_ Fraction of barleyDE in barleyortholist: 0.101374463586044
Expected overlap based on _1_ * _3_: 557.660924186828
wheat DE genes that have a DE barley ortholog, barley genes counted: 1105
_1_ barly DE genes that have a wheat ortholog, wheat genes counted: 2754
_2_ Fraction of orthogenes vs all, barley counted: 0.379687352413337
_3_ Fraction of wheatDE in wheatortholist: 0.284213447510308
Expected overlap based on _1_ * _3_: 782.723834443387
DE barley that have a DE wheat ortholog, wheat genes counted: 1617

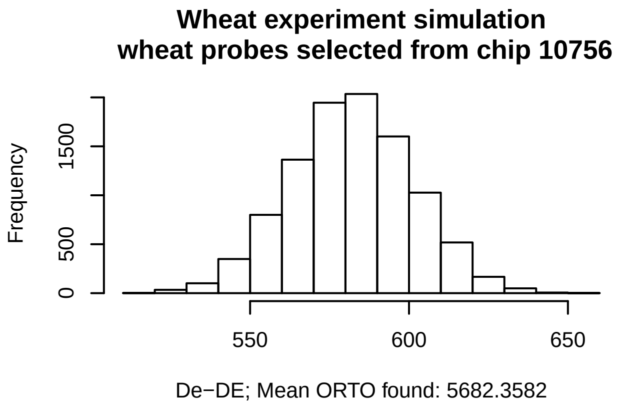


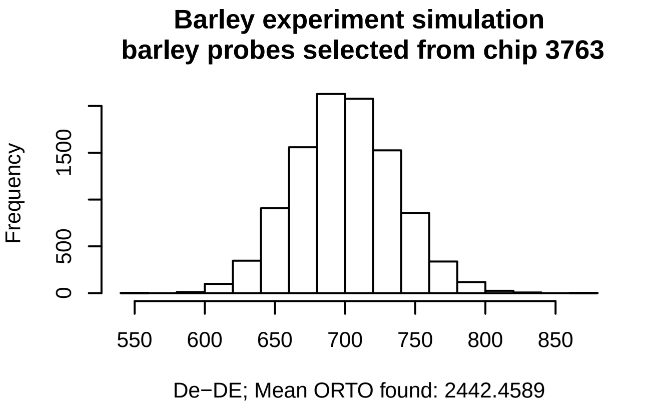


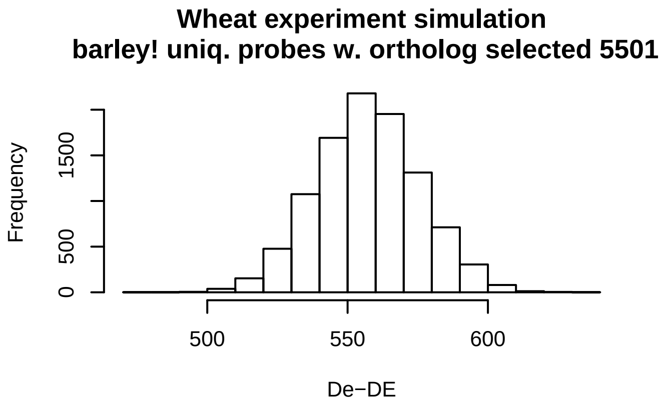


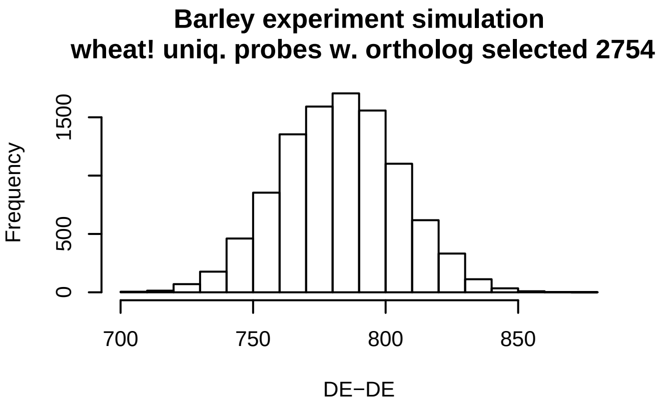

Supplement: Supplementary file 8 — Simulations of orthologue assignment within random wheat (barley) gene sets to general pathogen regulated barley (wheat) DEGs showing that the overlap found between wheat and barley general pathogen-regulated DEGs was larger than by chance. (PDF 10 kb) [file 12870_2017_1178_MOESM8_ESM.pdf]
