# Supplementary material for: A comparative analysis of nonhost resistance across the two Triticeae crop species wheat and barley
Source: BMC Plant Biol. 2017 Dec 4;17:232. doi: 10.1186/s12870-017-1178-0 (PMC5715502; doi:10.1186/s12870-017-1178-0)
Supplement: Supplementary file 10 — Numbers of genes found to be differentially regulated between host and nonhost interactions at different time points in wheat or barley after inoculation with adapted and non-adapted isolates of Blumeria, Magnaporthe and Puccinia (according to statistical analysis of microarray data). (PDF 51 kb) [file 12870_2017_1178_MOESM10_ESM.pdf]

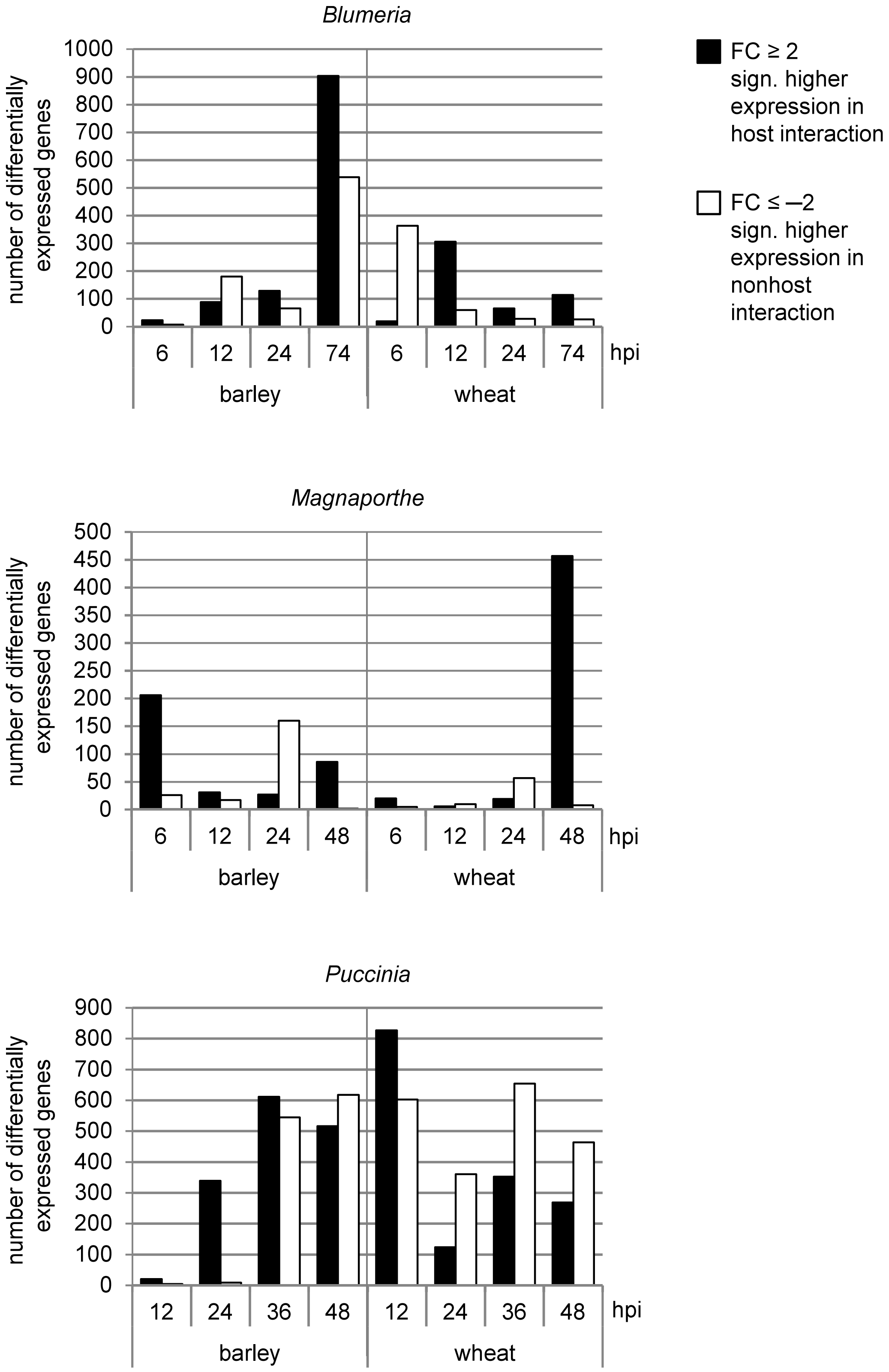

Figure S5. Numbers of genes found to be differentially regulated between host and nonhost interactions at different time points in wheat or barley after inoculation with adapted and non-adapted isolates of *Blumeria*, *Magnaporthe* and *Puccinia* (according to statistical analysis of microarray data)
