# Supplementary material for: A comparative analysis of nonhost resistance across the two Triticeae crop species wheat and barley
Source: BMC Plant Biol. 2017 Dec 4;17:232. doi: 10.1186/s12870-017-1178-0 (PMC5715502; doi:10.1186/s12870-017-1178-0)

B. graminis

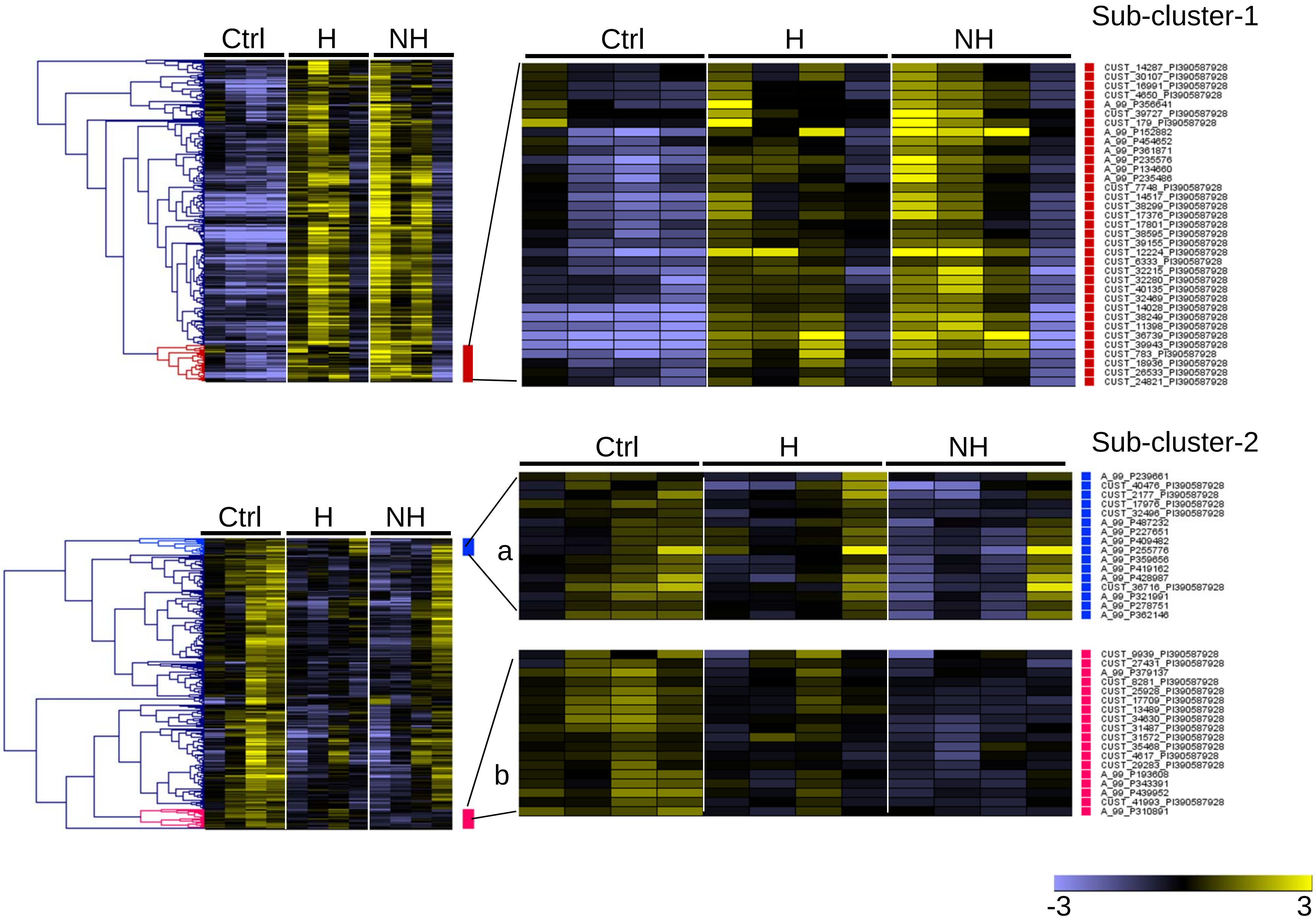
Ctrl
H
NH
Sub-cluster-1
Ctrl
H
NH
CUST_14287_PI390587928
CUST_30107_PI390587928
CUST_16991_PI390587928
CUST_4650_PI390587928
A_99_P356641
CUST_39727_PI390587928
CUST_179_PI390587928
A_99_P152882
A_99_P454652
A_99_P361871
A_99_P235576
A_99_P134660
A_99_P235486
CUST_7748_PI390587928
CUST_14517_PI390587928
CUST_38299_PI390587928
CUST_17376_PI390587928
CUST_17801_PI390587928
CUST_38595_PI390587928
CUST_39155_PI390587928
CUST_12224_PI390587928
CUST_6333_PI390587928
CUST_32215_PI390587928
CUST_32280_PI390587928
CUST_40135_PI390587928
CUST_32469_PI390587928
CUST_14028_PI390587928
CUST_38249_PI390587928
CUST_11398_PI390587928
CUST_36739_PI390587928
CUST_39943_PI390587928
CUST_783_PI390587928
CUST_18936_PI390587928
CUST_26533_PI390587928
CUST_24821_PI390587928
Ctrl
H
NH
Sub-cluster-2
Ctrl
H
NH
a
A_99_P239661
CUST_40476_PI390587928
CUST_2177_PI390587928
CUST_17976_PI390587928
CUST_32496_PI390587928
A_99_P487232
A_99_P227651
A_99_P409482
A_99_P255776
A_99_P359656
A_99_P419162
A_99_P428987
CUST_36716_PI390587928
A_99_P321991
A_99_P278751
A_99_P362146
b
CUST_9939_PI390587928
CUST_27431_PI390587928
A_99_P379137
CUST_8281_PI390587928
CUST_25928_PI390587928
CUST_17709_PI390587928
CUST_13489_PI390587928
CUST_34630_PI390587928
CUST_31487_PI390587928
CUST_31572_PI390587928
CUST_35468_PI390587928
CUST_4617_PI390587928
CUST_29283_PI390587928
A_99_P193608
A_99_P343391
A_99_P439952
CUST_41993_PI390587928
A_99_P310891
-3
3

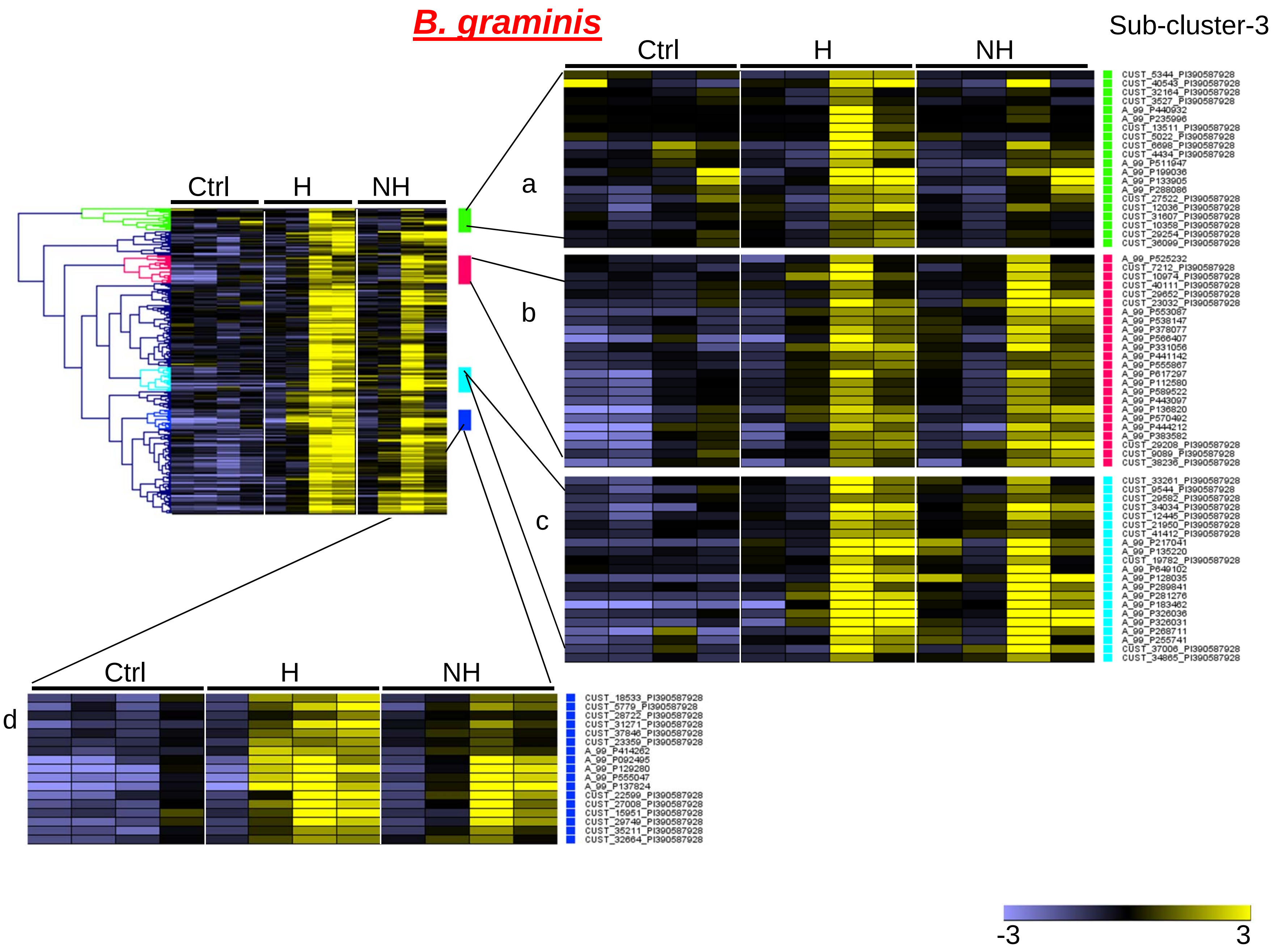

B. graminis
Sub-cluster-3
Ctrl
H
NH
a
b
c
d
CUST_5344_PI390587928
CUST_40543_PI390587928
CUST_32164_PI390587928
CUST_3527_PI390587928
A_99_P440932
A_99_P235996
CUST_13511_PI390587928
CUST_5022_PI390587928
CUST_6698_PI390587928
CUST_4434_PI390587928
A_99_P511947
A_99_P199036
A_99_P133905
A_99_P288086
CUST_27522_PI390587928
CUST_12036_PI390587928
CUST_31607_PI390587928
CUST_10358_PI390587928
CUST_29254_PI390587928
CUST_36099_PI390587928
A_99_P525232
CUST_7212_PI390587928
CUST_10974_PI390587928
CUST_40111_PI390587928
CUST_29652_PI390587928
CUST_23032_PI390587928
A_99_P553087
A_99_P538147
A_99_P378077
A_99_P566407
A_99_P331056
A_99_P441142
A_99_P555867
A_99_P617297
A_99_P112580
A_99_P589522
A_99_P443097
A_99_P136820
A_99_P570492
A_99_P444212
A_99_P383582
CUST_29208_PI390587928
CUST_9089_PI390587928
CUST_38236_PI390587928
CUST_33261_PI390587928
CUST_9544_PI390587928
CUST_29582_PI390587928
CUST_34034_PI390587928
CUST_12445_PI390587928
CUST_21950_PI390587928
CUST_41412_PI390587928
A_99_P217041
A_99_P135220
CUST_19782_PI390587928
A_99_P649102
A_99_P128035
A_99_P289841
A_99_P281276
A_99_P183462
A_99_P326036
A_99_P326031
A_99_P268711
A_99_P255741
CUST_37006_PI390587928
CUST_34865_PI390587928
CUST_18533_PI390587928
CUST_5779_PI390587928
CUST_28722_PI390587928
CUST_31271_PI390587928
CUST_37846_PI390587928
CUST_23359_PI390587928
A_99_P414262
A_99_P092495
A_99_P129280
A_99_P555047
A_99_P137824
CUST_22599_PI390587928
CUST_27008_PI390587928
CUST_15951_PI390587928
CUST_29749_PI390587928
CUST_35211_PI390587928
CUST_32664_PI390587928
-3
3

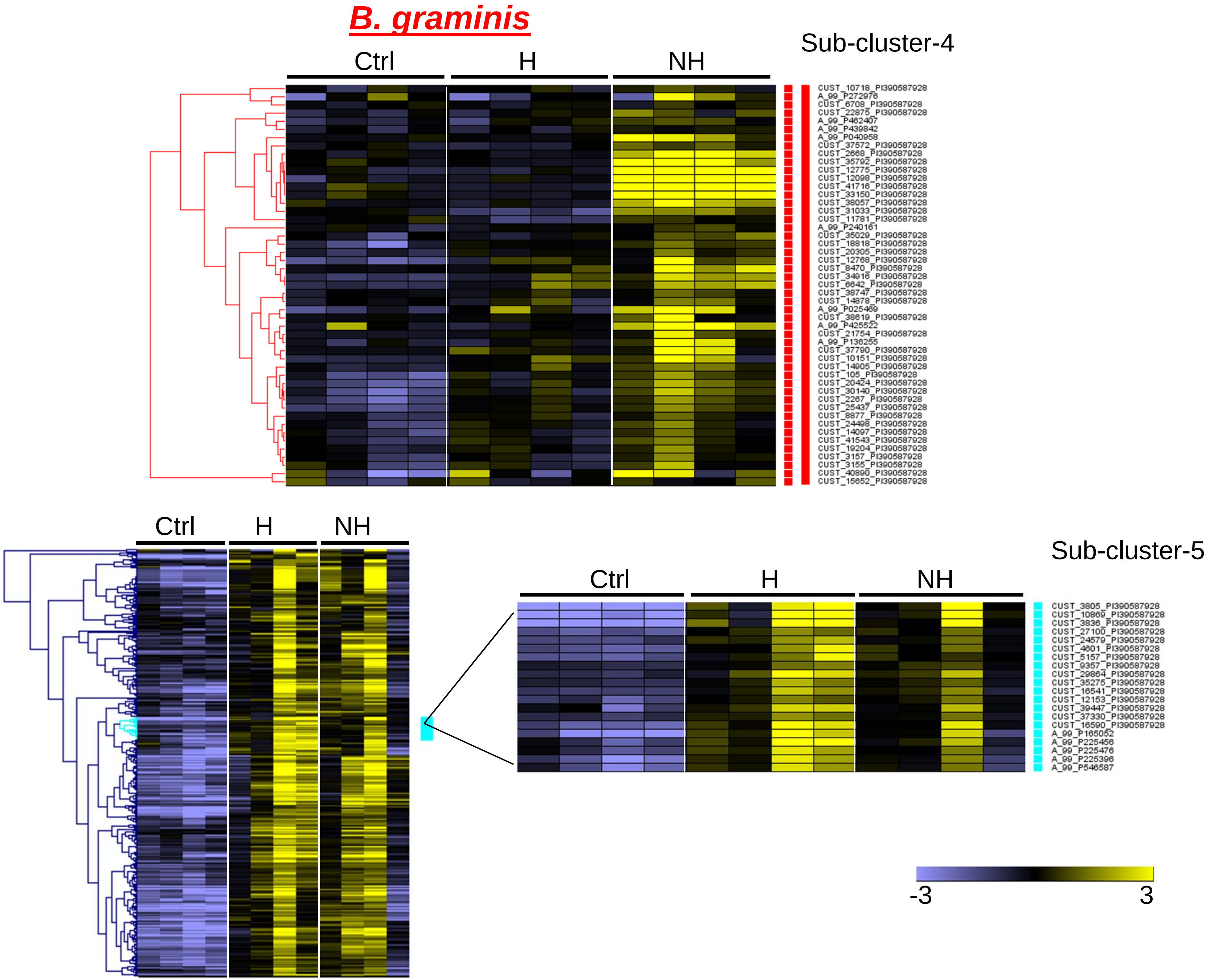
B. graminis
Sub-cluster-4
Ctrl
H
NH
CUST_10718_PI390587928
A_99_P272976
CUST_6708_PI390587928
CUST_22875_PI390587928
A_99_P462407
A_99_P439842
A_99_P040958
CUST_37572_PI390587928
CUST_2668_PI390587928
CUST_35792_PI390587928
CUST_12775_PI390587928
CUST_12098_PI390587928
CUST_41716_PI390587928
CUST_33150_PI390587928
CUST_38057_PI390587928
CUST_31033_PI390587928
CUST_11781_PI390587928
A_99_P240161
CUST_35029_PI390587928
CUST_18818_PI390587928
CUST_20305_PI390587928
CUST_12768_PI390587928
CUST_8470_PI390587928
CUST_34916_PI390587928
CUST_6642_PI390587928
CUST_38747_PI390587928
CUST_14878_PI390587928
A_99_P025469
CUST_38619_PI390587928
A_99_P425522
CUST_21754_PI390587928
A_99_P136255
CUST_37790_PI390587928
CUST_10151_PI390587928
CUST_14905_PI390587928
CUST_105_PI390587928
CUST_20424_PI390587928
CUST_30140_PI390587928
CUST_2267_PI390587928
CUST_25437_PI390587928
CUST_8877_PI390587928
CUST_24498_PI390587928
CUST_14097_PI390587928
CUST_41543_PI390587928
CUST_19204_PI390587928
CUST_3157_PI390587928
CUST_3155_PI390587928
CUST_40890_PI390587928
CUST_15652_PI390587928
Ctrl
H
NH
Sub-cluster-5
Ctrl
H
NH
CUST_3805_PI390587928
CUST_10869_PI390587928
CUST_3836_PI390587928
CUST_27100_PI390587928
CUST_24579_PI390587928
CUST_4601_PI390587928
CUST_5157_PI390587928
CUST_9357_PI390587928
CUST_29864_PI390587928
CUST_35275_PI390587928
CUST_16541_PI390587928
CUST_12153_PI390587928
CUST_39447_PI390587928
CUST_37330_PI390587928
CUST_16590_PI390587928
A_99_P165052
A_99_P225456
A_99_P225476
A_99_P225396
A_99_P546587
-3
3

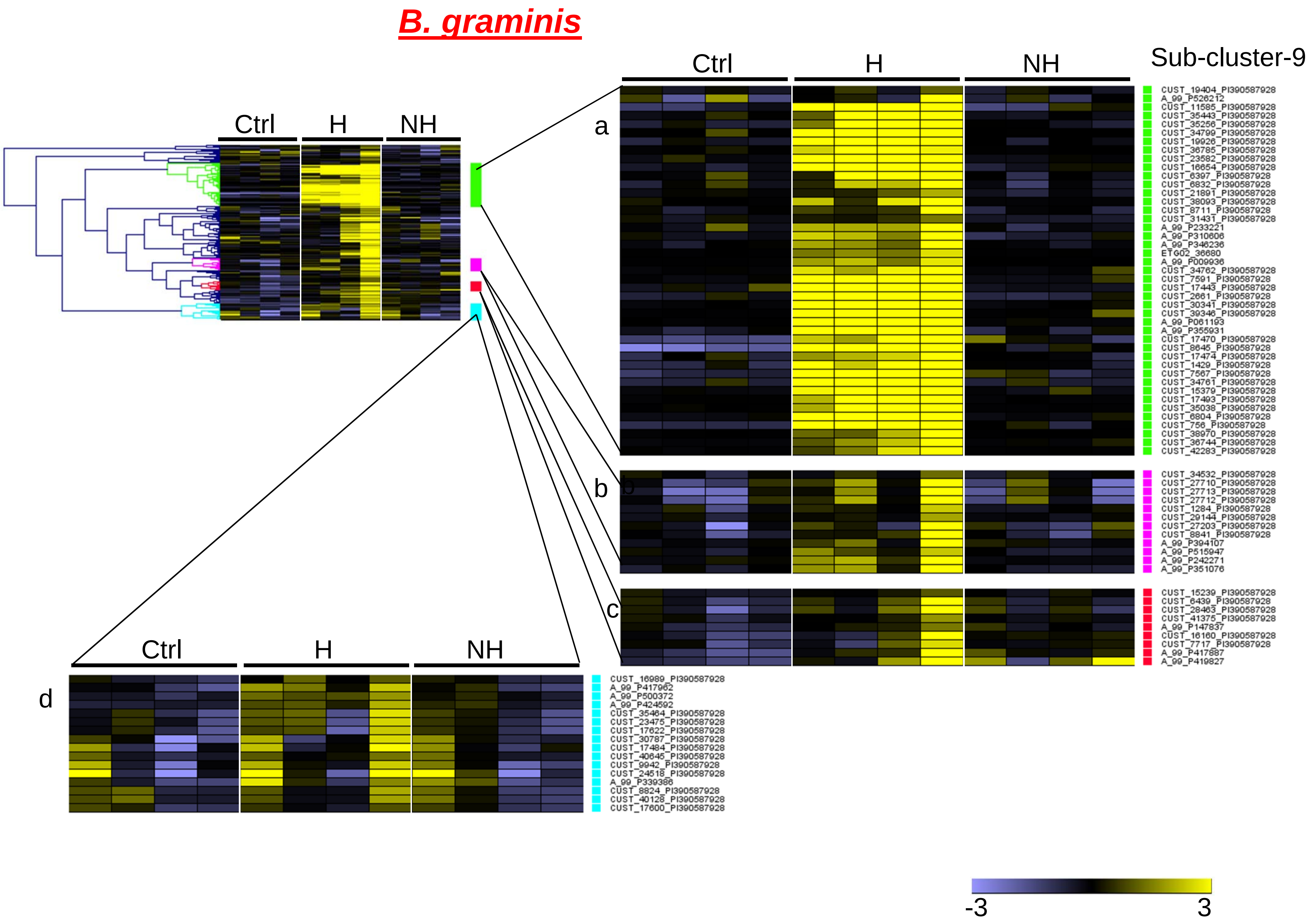

B. graminis
Sub-cluster-9
Ctrl
H
NH
a
b
c
d
CUST_19404_PI390587928
A_99_P526212
CUST_11585_PI390587928
CUST_35443_PI390587928
CUST_35256_PI390587928
CUST_34799_PI390587928
CUST_19926_PI390587928
CUST_36785_PI390587928
CUST_23582_PI390587928
CUST_16654_PI390587928
CUST_6397_PI390587928
CUST_6832_PI390587928
CUST_21891_PI390587928
CUST_38093_PI390587928
CUST_8711_PI390587928
CUST_31431_PI390587928
A_99_P233221
A_99_P310606
A_99_P346236
ETG02_36680
A_99_P009936
CUST_34762_PI390587928
CUST_7591_PI390587928
CUST_17443_PI390587928
CUST_2661_PI390587928
CUST_30341_PI390587928
CUST_39346_PI390587928
A_99_P061193
A_99_P355931
CUST_17470_PI390587928
CUST_8645_PI390587928
CUST_17474_PI390587928
CUST_1429_PI390587928
CUST_7567_PI390587928
CUST_34761_PI390587928
CUST_15379_PI390587928
CUST_17493_PI390587928
CUST_35038_PI390587928
CUST_6804_PI390587928
CUST_756_PI390587928
CUST_38970_PI390587928
CUST_36744_PI390587928
CUST_42283_PI390587928
CUST_34532_PI390587928
CUST_27710_PI390587928
CUST_27713_PI390587928
CUST_27712_PI390587928
CUST_1284_PI390587928
CUST_29144_PI390587928
CUST_27203_PI390587928
CUST_8841_PI390587928
A_99_P394107
A_99_P515947
A_99_P242271
A_99_P351076
CUST_15239_PI390587928
CUST_6439_PI390587928
CUST_28463_PI390587928
CUST_41375_PI390587928
A_99_P147837
CUST_16160_PI390587928
CUST_7717_PI390587928
A_99_P417887
A_99_P419827
CUST_16989_PI390587928
A_99_P417962
A_99_P500372
A_99_P424592
CUST_35464_PI390587928
CUST_23475_PI390587928
CUST_17622_PI390587928
CUST_30787_PI390587928
CUST_17484_PI390587928
CUST_40645_PI390587928
CUST_9942_PI390587928
CUST_24518_PI390587928
A_99_P339386
CUST_8824_PI390587928
CUST_40128_PI390587928
CUST_17600_PI390587928
-3
3

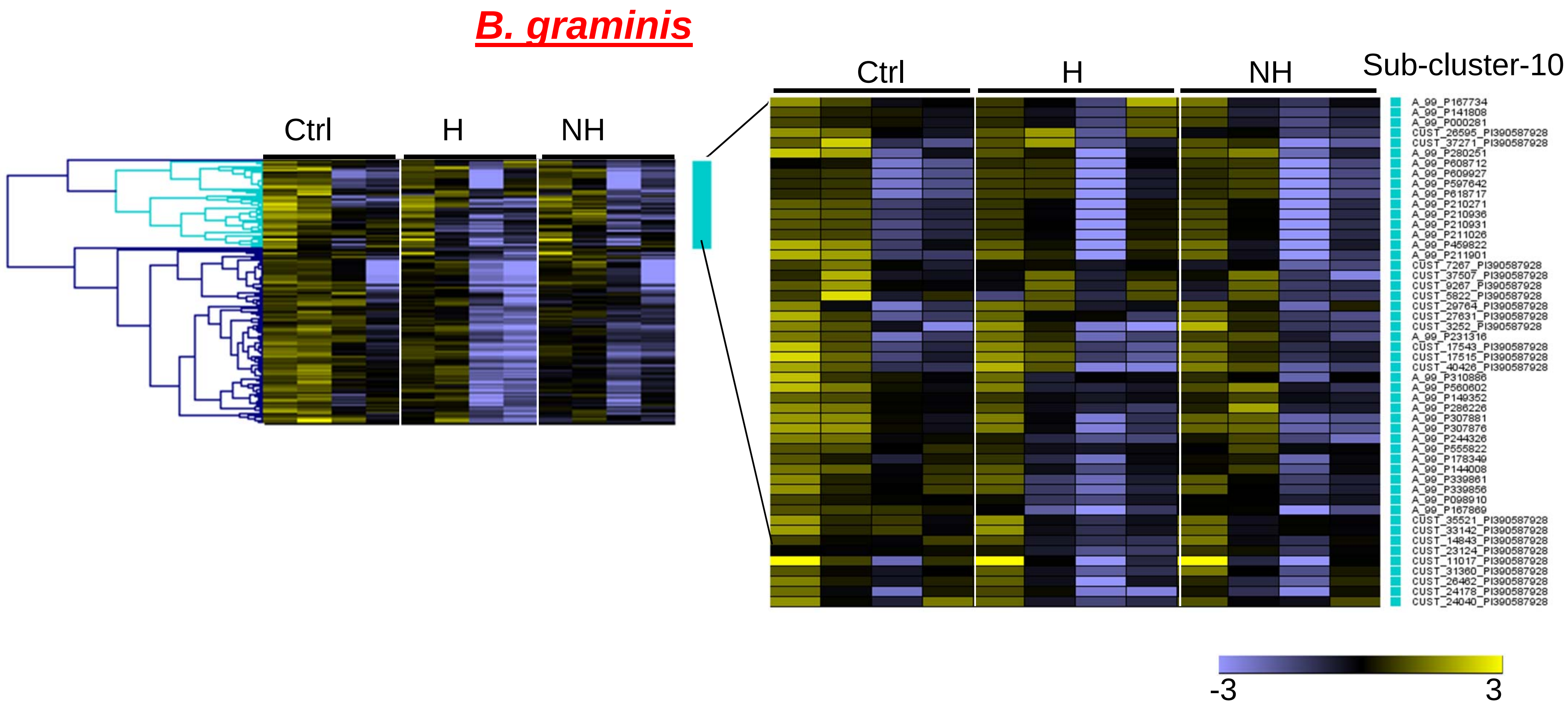
B. graminis
Ctrl
H
NH
Ctrl
H
NH
Sub-cluster-10
A_99_P167734
A_99_P141808
A_99_P000281
CUST_26595_PI390587928
CUST_37271_PI390587928
A_99_P280251
A_99_P608712
A_99_P609927
A_99_P597642
A_99_P618717
A_99_P210271
A_99_P210936
A_99_P210931
A_99_P211026
A_99_P459822
A_99_P211901
CUST_7267_PI390587928
CUST_37507_PI390587928
CUST_9267_PI390587928
CUST_5822_PI390587928
CUST_29764_PI390587928
CUST_27631_PI390587928
CUST_3252_PI390587928
A_99_P231316
CUST_17543_PI390587928
CUST_17515_PI390587928
CUST_40426_PI390587928
A_99_P310886
A_99_P560602
A_99_P149352
A_99_P286226
A_99_P307881
A_99_P307876
A_99_P244326
A_99_P555822
A_99_P178349
A_99_P144008
A_99_P339861
A_99_P339856
A_99_P098910
A_99_P167869
CUST_35521_PI390587928
CUST_33142_PI390587928
CUST_14843_PI390587928
CUST_23124_PI390587928
CUST_11017_PI390587928
CUST_31360_PI390587928
CUST_26462_PI390587928
CUST_24178_PI390587928
CUST_24040_PI390587928
-3
3

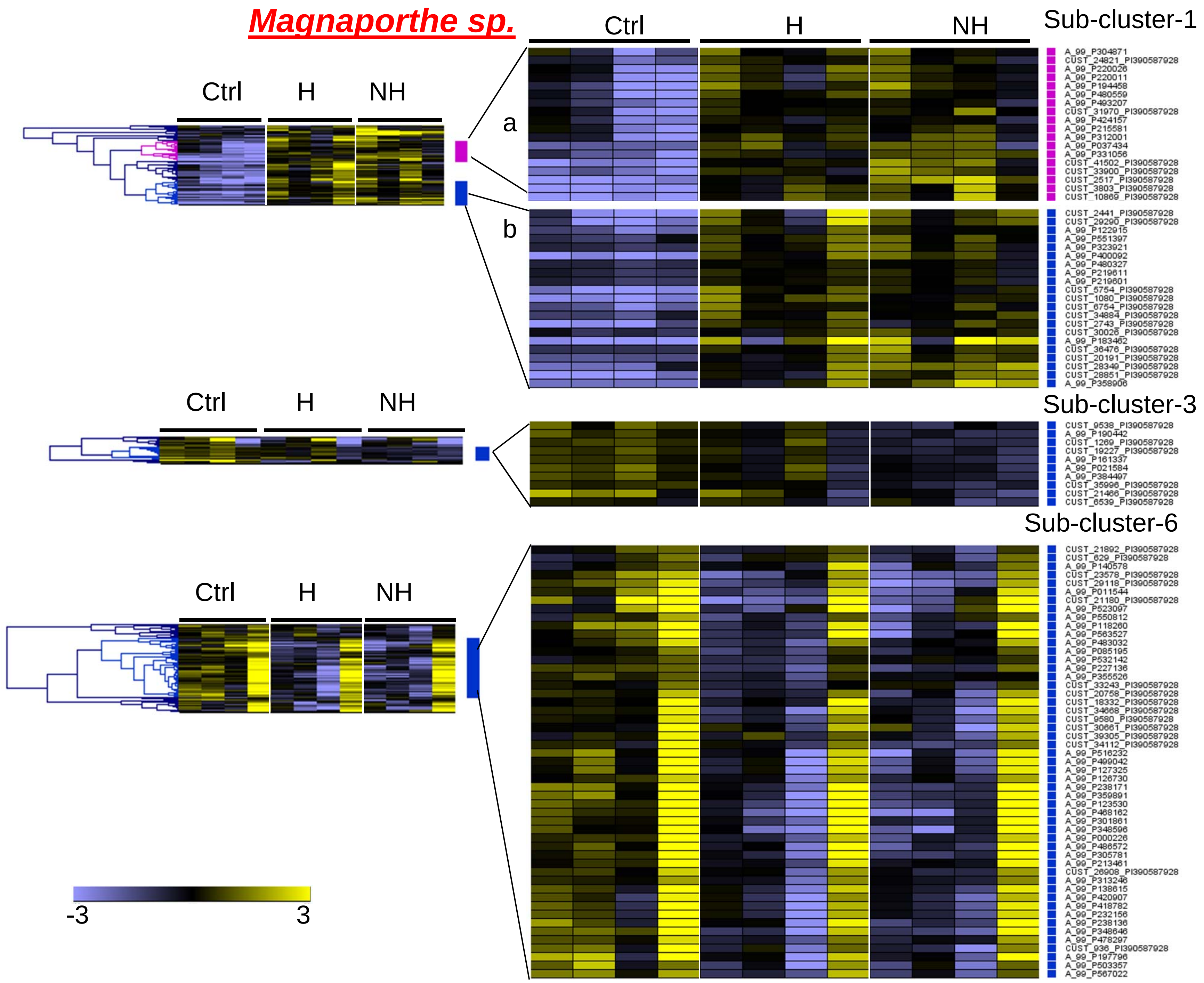

Magnaporthe sp.
Ctrl
H
NH
Sub-cluster-1
a
b
A_99_P304871
CUST_24821_PI390587928
A_99_P220026
A_99_P220011
A_99_P194458
A_99_P480559
A_99_P493207
CUST_31970_PI390587928
A_99_P424157
A_99_P215581
A_99_P312001
A_99_P037434
A_99_P331056
CUST_41502_PI390587928
CUST_33900_PI390587928
CUST_2517_PI390587928
CUST_3803_PI390587928
CUST_10869_PI390587928
CUST_2441_PI390587928
CUST_29290_PI390587928
A_99_P122915
A_99_P551397
A_99_P323921
A_99_P400092
A_99_P480327
A_99_P219611
A_99_P219601
CUST_5754_PI390587928
CUST_1080_PI390587928
CUST_6754_PI390587928
CUST_34884_PI390587928
CUST_2743_PI390587928
CUST_30026_PI390587928
A_99_P183462
CUST_36476_PI390587928
CUST_20191_PI390587928
CUST_28349_PI390587928
CUST_28851_PI390587928
A_99_P358906
Sub-cluster-3
CUST_9538_PI390587928
A_99_P190442
CUST_1269_PI390587928
CUST_19227_PI390587928
A_99_P161337
A_99_P021584
A_99_P384497
CUST_35996_PI390587928
CUST_21466_PI390587928
CUST_6539_PI390587928
Sub-cluster-6
CUST_21892_PI390587928
CUST_629_PI390587928
A_99_P140578
CUST_23578_PI390587928
CUST_29118_PI390587928
A_99_P011544
CUST_21180_PI390587928
A_99_P523097
A_99_P550812
A_99_P118260
A_99_P563527
A_99_P483032
A_99_P085195
A_99_P532142
A_99_P227136
A_99_P355526
CUST_33243_PI390587928
CUST_20758_PI390587928
CUST_18332_PI390587928
CUST_34668_PI390587928
CUST_9580_PI390587928
CUST_30661_PI390587928
CUST_39305_PI390587928
CUST_34112_PI390587928
A_99_P516232
A_99_P499042
A_99_P127325
A_99_P126730
A_99_P238171
A_99_P359891
A_99_P123530
A_99_P468162
A_99_P301861
A_99_P348596
A_99_P000226
A_99_P486572
A_99_P305781
A_99_P213461
CUST_26908_PI390587928
A_99_P313246
A_99_P138615
A_99_P420907
A_99_P418782
A_99_P232156
A_99_P238136
A_99_P348646
A_99_P478297
CUST_936_PI390587928
A_99_P197796
A_99_P503357
A_99_P567022
-3
3

Magnaporthe sp.

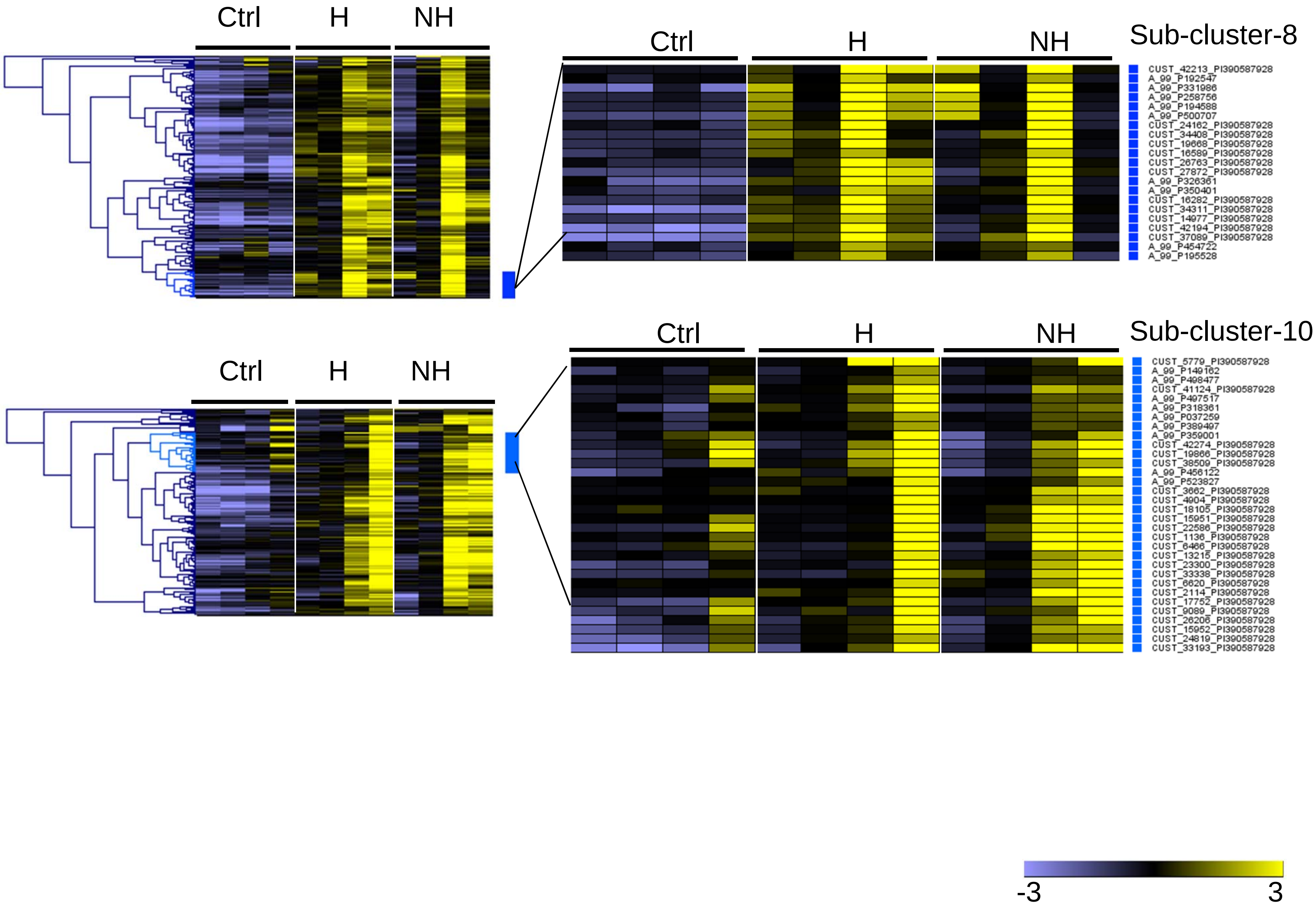

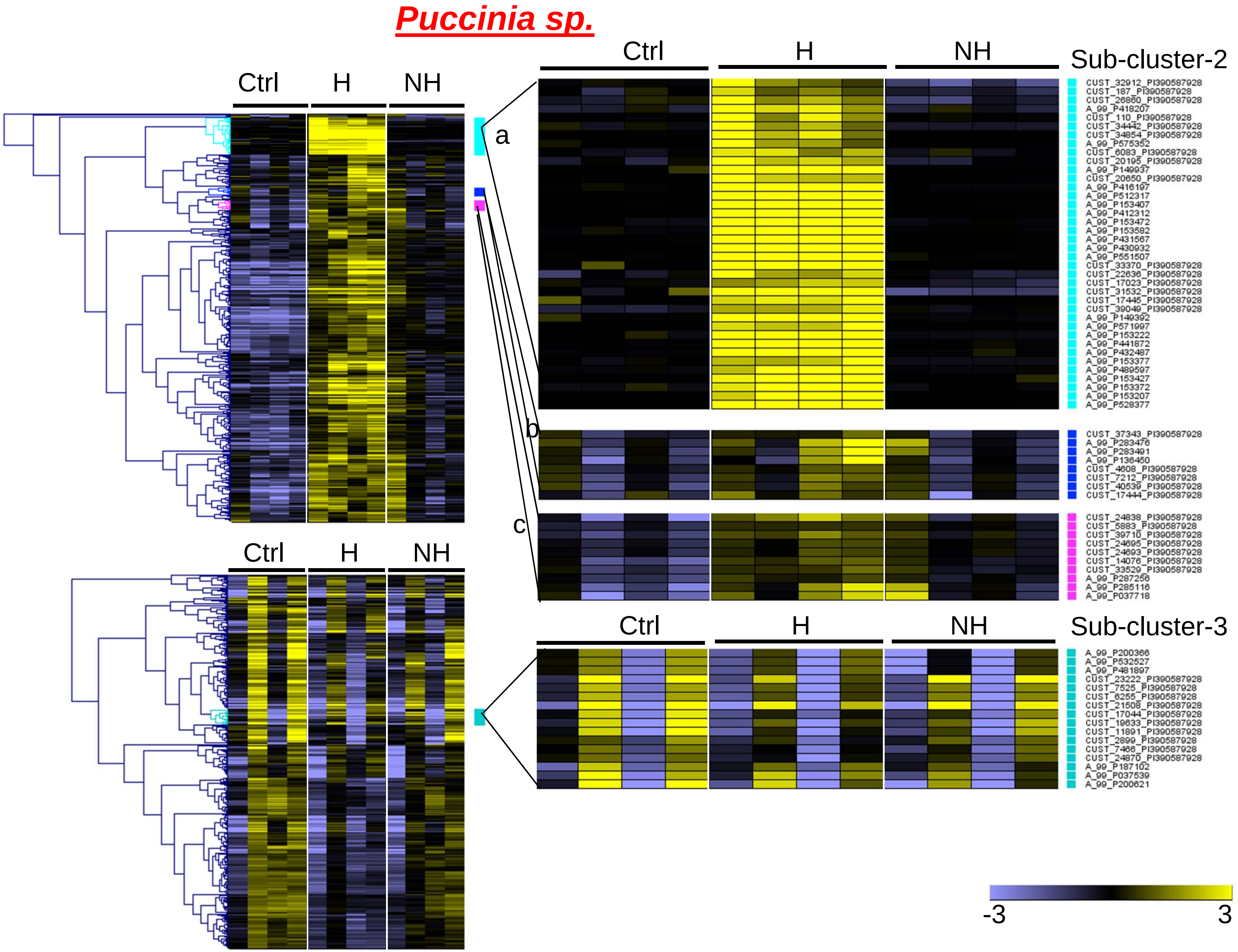
Puccinia sp.
Ctrl
H
NH
a
b
c
Ctrl
H
NH
Ctrl
H
NH
Sub-cluster-2
CUST_32912_PI390587928
CUST_187_PI390587928
CUST_26860_PI390587928
A_99_P418207
CUST_110_PI390587928
CUST_34442_PI390587928
CUST_34854_PI390587928
A_99_P575352
CUST_6083_PI390587928
CUST_20195_PI390587928
A_99_P149937
CUST_20650_PI390587928
A_99_P416197
A_99_P512317
A_99_P153407
A_99_P412312
A_99_P153472
A_99_P153582
A_99_P431567
A_99_P430932
A_99_P551507
CUST_33370_PI390587928
CUST_22636_PI390587928
CUST_17023_PI390587928
CUST_31532_PI390587928
CUST_17445_PI390587928
CUST_39049_PI390587928
A_99_P149392
A_99_P571997
A_99_P153222
A_99_P441872
A_99_P432487
A_99_P153377
A_99_P489597
A_99_P153427
A_99_P153372
A_99_P153207
A_99_P528377
CUST_37343_PI390587928
A_99_P283476
A_99_P283491
A_99_P136450
CUST_4608_PI390587928
CUST_7212_PI390587928
CUST_40539_PI390587928
CUST_17444_PI390587928
CUST_24838_PI390587928
CUST_5883_PI390587928
CUST_39710_PI390587928
CUST_24695_PI390587928
CUST_24693_PI390587928
CUST_14076_PI390587928
CUST_33529_PI390587928
A_99_P287256
A_99_P285116
A_99_P037718
Ctrl
H
NH
Sub-cluster-3
A_99_P200366
A_99_P532527
A_99_P481897
CUST_23222_PI390587928
CUST_7525_PI390587928
CUST_6255_PI390587928
CUST_21508_PI390587928
CUST_17044_PI390587928
CUST_19633_PI390587928
CUST_11891_PI390587928
CUST_2899_PI390587928
CUST_7466_PI390587928
CUST_24870_PI390587928
A_99_P187102
A_99_P037539
A_99_P200621
-3
3

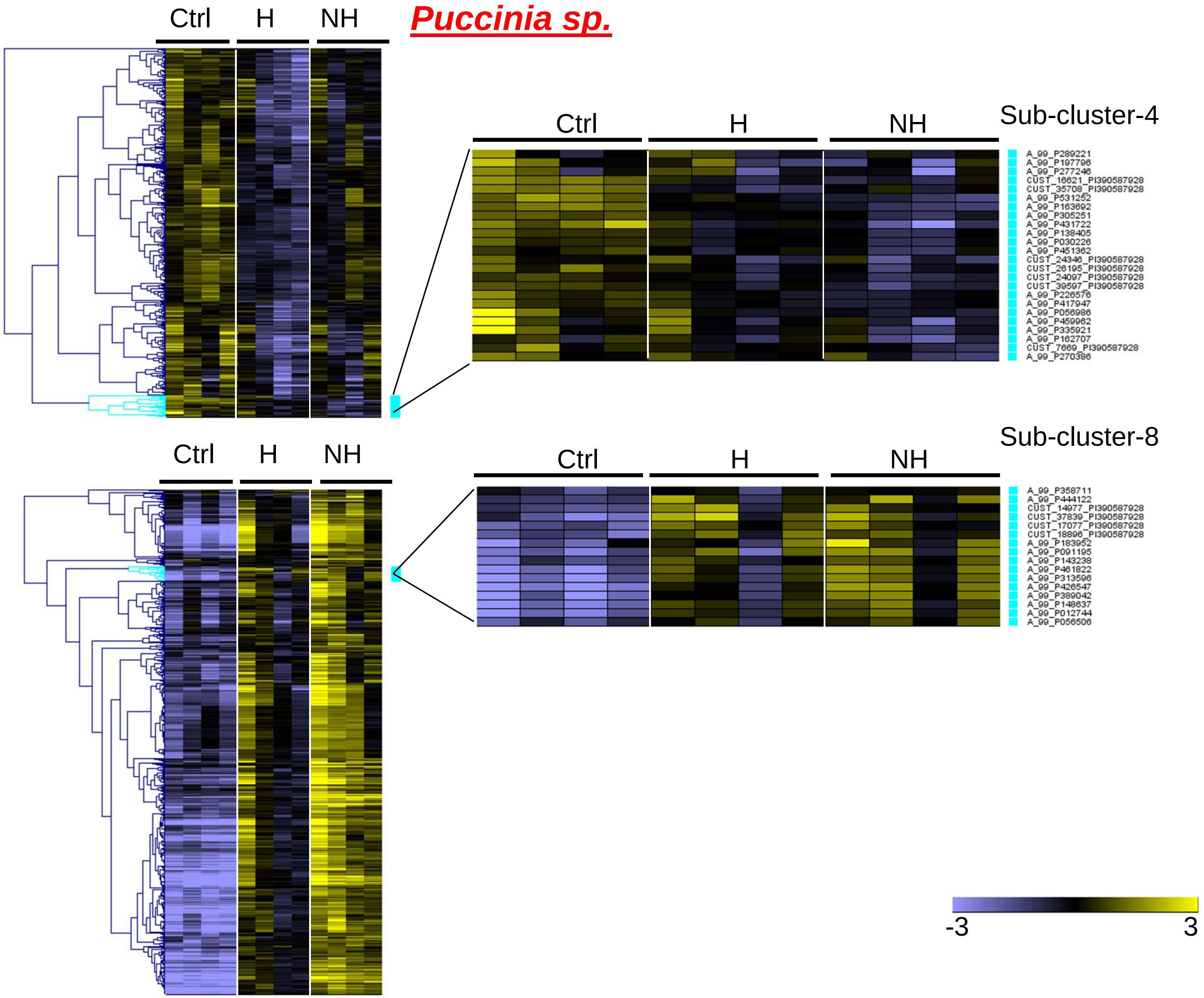

Ctrl
H
NH
Puccinia sp.
Sub-cluster-4
Ctrl
H
NH
A_99_P289221
A_99_P197796
A_99_P277246
CUST_16621_PI390587928
CUST_35708_PI390587928
A_99_P531252
A_99_P163692
A_99_P305251
A_99_P431722
A_99_P138405
A_99_P030226
A_99_P451362
CUST_24346_PI390587928
CUST_26195_PI390587928
CUST_24097_PI390587928
CUST_39597_PI390587928
A_99_P226576
A_99_P417947
A_99_P056986
A_99_P459962
A_99_P335921
A_99_P162707
CUST_7669_PI390587928
A_99_P270386
Ctrl
H
NH
Sub-cluster-8
Ctrl
H
NH
A_99_P358711
A_99_P444122
CUST_14977_PI390587928
CUST_37839_PI390587928
CUST_17077_PI390587928
CUST_18896_PI390587928
A_99_P183952
A_99_P091195
A_99_P143238
A_99_P461822
A_99_P313596
A_99_P426547
A_99_P389042
A_99_P148637
A_99_P012744
A_99_P056506
-3
3

Puccinia sp.

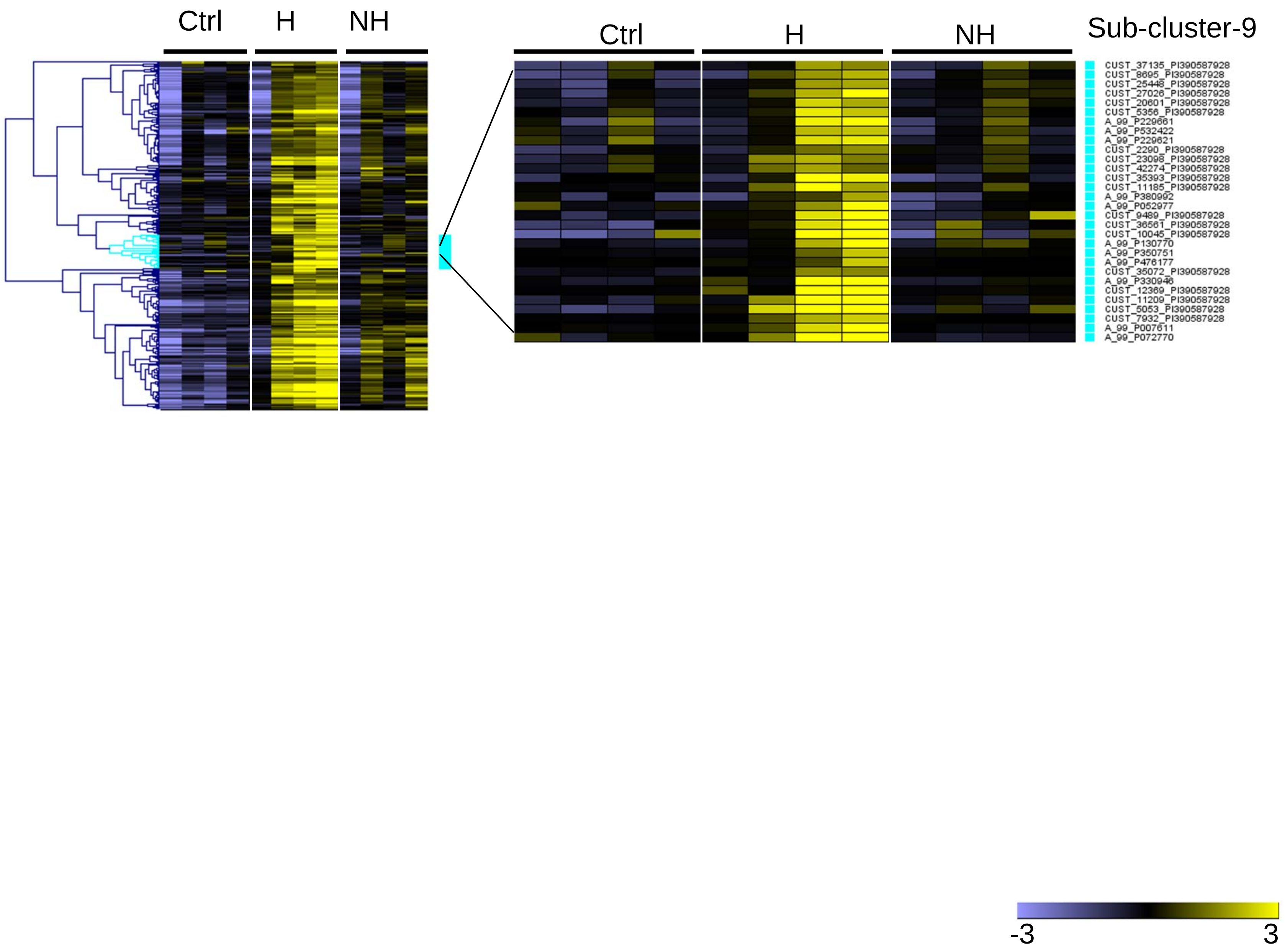

Ctrl
H
NH
Ctrl
H
NH
Sub-cluster-9
CUST_37135_PI390587928
CUST_8695_PI390587928
CUST_25448_PI390587928
CUST_27026_PI390587928
CUST_20601_PI390587928
CUST_5356_PI390587928
A_99_P229661
A_99_P532422
A_99_P229621
CUST_2290_PI390587928
CUST_23098_PI390587928
CUST_42274_PI390587928
CUST_35393_PI390587928
CUST_11185_PI390587928
A_99_P380992
A_99_P052977
CUST_9489_PI390587928
CUST_36561_PI390587928
CUST_10045_PI390587928
A_99_P130770
A_99_P350751
A_99_P476177
CUST_35072_PI390587928
A_99_P330946
CUST_12369_PI390587928
CUST_11209_PI390587928
CUST_5053_PI390587928
CUST_7932_PI390587928
A_99_P007611
A_99_P072770
-3
3

Puccinia sp.

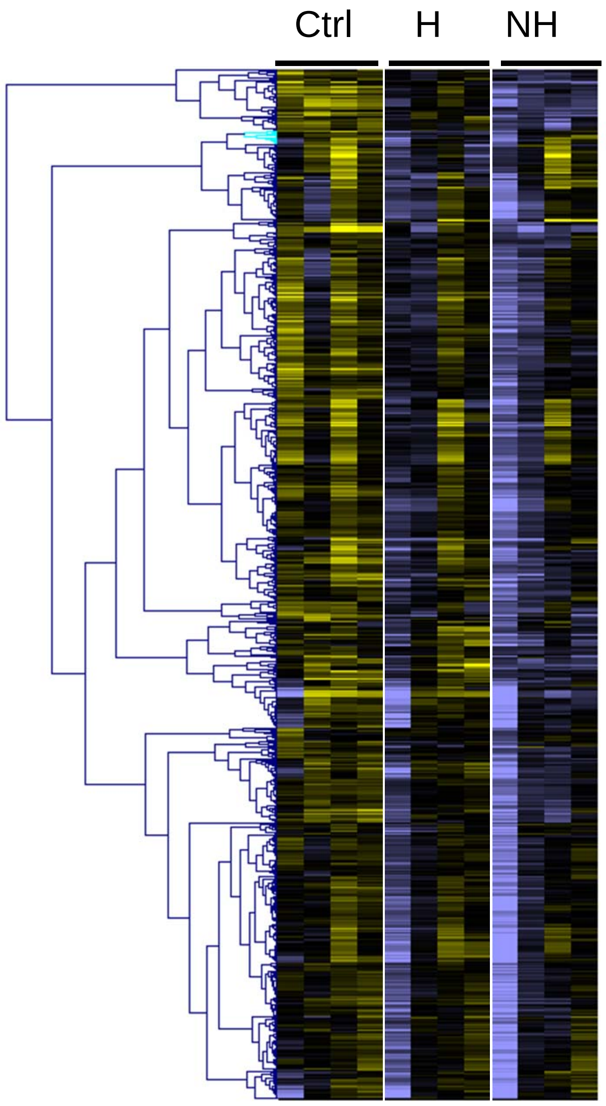

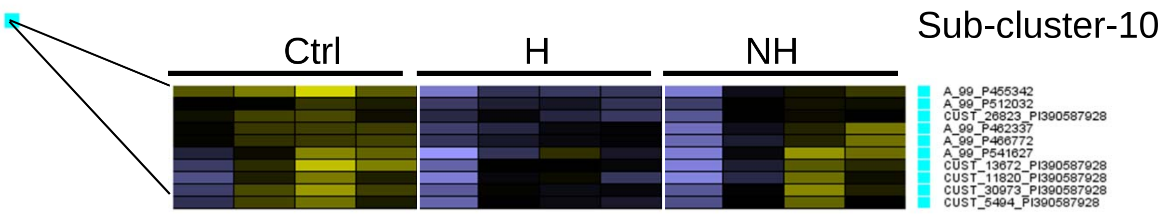

Figure S6. Results of *k*-means clustering followed by hierarchical clustering on the nonhost-related DEGs of wheat and barley in each pathosystem.

In an independent approach to identify co-regulated orthologues, a *k*-means clustering followed by a hierarchical clustering of individual *k*-means cluster members was carried out for both cereal species and respective interactions. For this, the normalized signal intensity values of host and nonhost DEGs (Fig. 2b) of the respective pathosystem were median-centered separately for wheat and barley in Genesis software (release 1.8.0, [82]). The median-centered signal values of both species were pooled for each pathosystem to form a single list, and a *k*-means clustering analysis was done in Genesis with default settings. Individual *k*-means clusters were then hierarchical clustered in Genesis with Pearson correlation, average linkage settings. To identify co-regulated regulons, sub-clusters with the occurrence of both wheat and barley probes (left clustering image; nodes with sub-clusters highlighted in color) were manually identified and extracted. A zoomed in image of these sub-clusters is shown on the right. Please note the occurrence of both wheat (A_99_) and barley (CUST_) probe IDs in the same node. In order to determine the gene function relation among these co-regulated wheat and barley DEGs, MapMan (3.5.1R2) and BlastN analyses (default parameters, BLASTN 2.2.29+) were carried out.

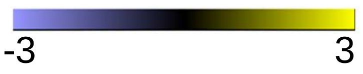

Supplement: Supplementary file 11 — Results of k-means clustering followed by hierarchical clustering on the nonhost-related DEGs of wheat and barley in each pathosystem. In an independent approach to identify co-regulated orthologues, a k-means clustering followed by a hierarchical clustering of individual k-means cluster members was carried out for both cereal species and respective interactions. For this, the normalized signal intensity values of host and nonhost DEGs (Fig. 2b) of the respective pathosystem were median-centered separately for wheat and barley in Genesis software (release 1.8.0, [88]). The median-centered signal values of both species were pooled for each pathosystem to form a single list, and a k-means clustering analysis was done in Genesis with default settings. Individual k-means clusters were then hierarchical clustered in Genesis with Pearson correlation, average linkage settings. To identify co-regulated regulons, sub-clusters with the occurrence of both wheat and barley probes (left clustering image; nodes with sub-clusters highlighted in color) were manually identified and extracted. A zoomed in image of these sub-clusters is shown on the right. Please note the occurrence of both wheat (A_99_) and barley (CUST_) probe IDs in the same node. In order to determine the gene function relation among these co-regulated wheat and barley DEGs, MapMan (3.5.1R2) and BlastN analyses (default parameters, BLASTN 2.2.29+) were carried out. (PDF 3400 kb) [file 12870_2017_1178_MOESM11_ESM.pdf]
