# Supplementary material for: A comparative analysis of nonhost resistance across the two Triticeae crop species wheat and barley
Source: BMC Plant Biol. 2017 Dec 4;17:232. doi: 10.1186/s12870-017-1178-0 (PMC5715502; doi:10.1186/s12870-017-1178-0)
Supplement: Supplementary file 13 — Overrepresentation analysis of functional MapMan categories within the intersections of general pathogen regulated wheat and barley DEGs (a) and DEGs with differential expression between host and nonhost interaction (b) found for the three pathosystems Blumeria, Magnaporthe and Puccinia. Over- or underrepresentation and statistical significance according to Fisher Exact Test were calculated with MapMan ORA tool [35, 36]. All probes assigned to MapMan BINs were taken as reference. The log ratio of presentation in the gene set and in the reference is shown for BINs found to be significant (BINs comprising 5 or less probes were excluded). (PDF 14 kb) [file 12870_2017_1178_MOESM13_ESM.pdf]

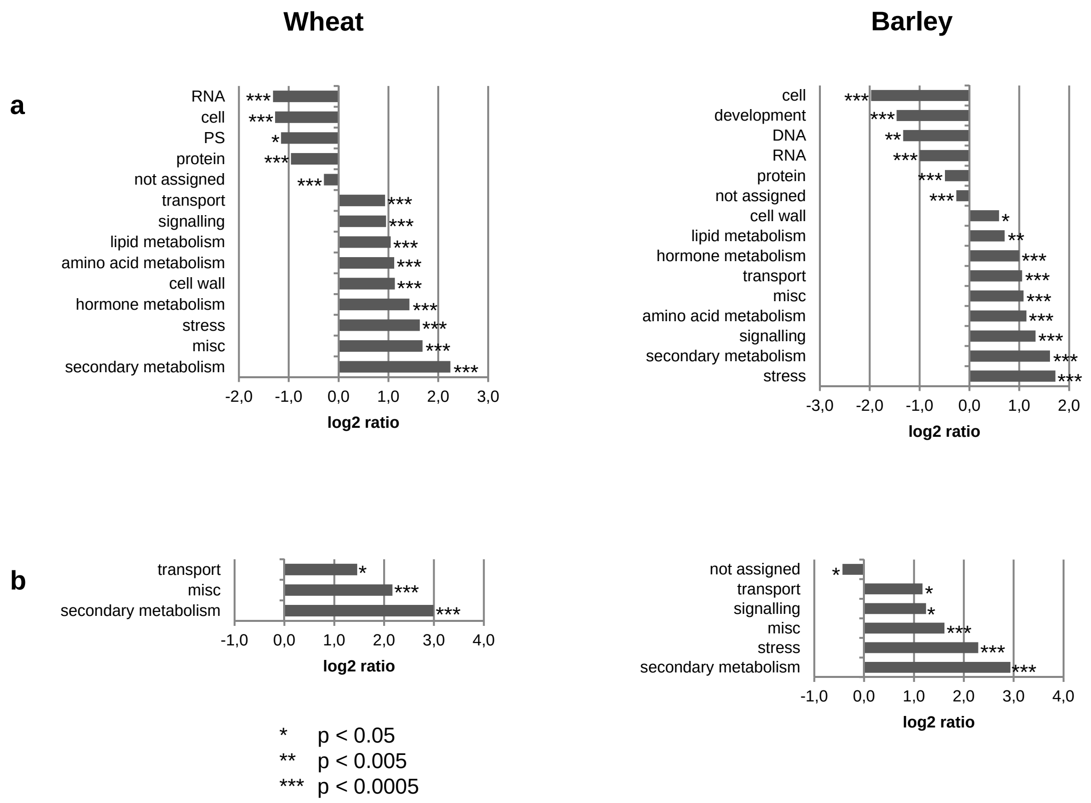

Figure S7. Overrepresentation analysis of functional MapMan categories within the intersections of general pathogen regulated wheat and barley DEGs (**a**) and DEGs with differential expression between host and nonhost interaction (**b**) found for the three pathosystems *Blumeria, Magnaporthe* and *Puccinia*. Over- or underrepresentation and statistical significance according to Fisher Exact Test were calculated with MapMan ORA tool [35,36]. All probes assigned to MapMan BINs were taken as reference. The log ratio of presentation in the gene set and in the reference is shown for BINs found to be significant (BINs comprising 5 or less probes were excluded).
