# Supplementary material for: A comparative analysis of nonhost resistance across the two Triticeae crop species wheat and barley
Source: BMC Plant Biol. 2017 Dec 4;17:232. doi: 10.1186/s12870-017-1178-0 (PMC5715502; doi:10.1186/s12870-017-1178-0)
Supplement: Supplementary file 14 — Clusterings of Puccinia microarray data leading to the decision to exclude one of three biological barley replicates. Ideally, the replications (1, 2, 3) from the same treatment (mock, host, nonhost) and time point (12, 24, 36 and 48 hpi) should cluster together. The clustering of chips was very clear in case of wheat/Puccinia interaction (a). In case of barley/Puccinia this was not the case: 7 out of 12 chips from replication 1 did not cluster into their respective subclades (b) (coloured arrows indicate chips out of their respective clusters). Therefore the complete replication 1 of barley/Puccinia interactions was omitted from the final analysis, resulting in a great enrichment of host/nonhost differentially regulated genes (11 according to analysis with all 3 replicates, 1824 according to analysis without replicate 1). (PDF 1063 kb) [file 12870_2017_1178_MOESM14_ESM.pdf]

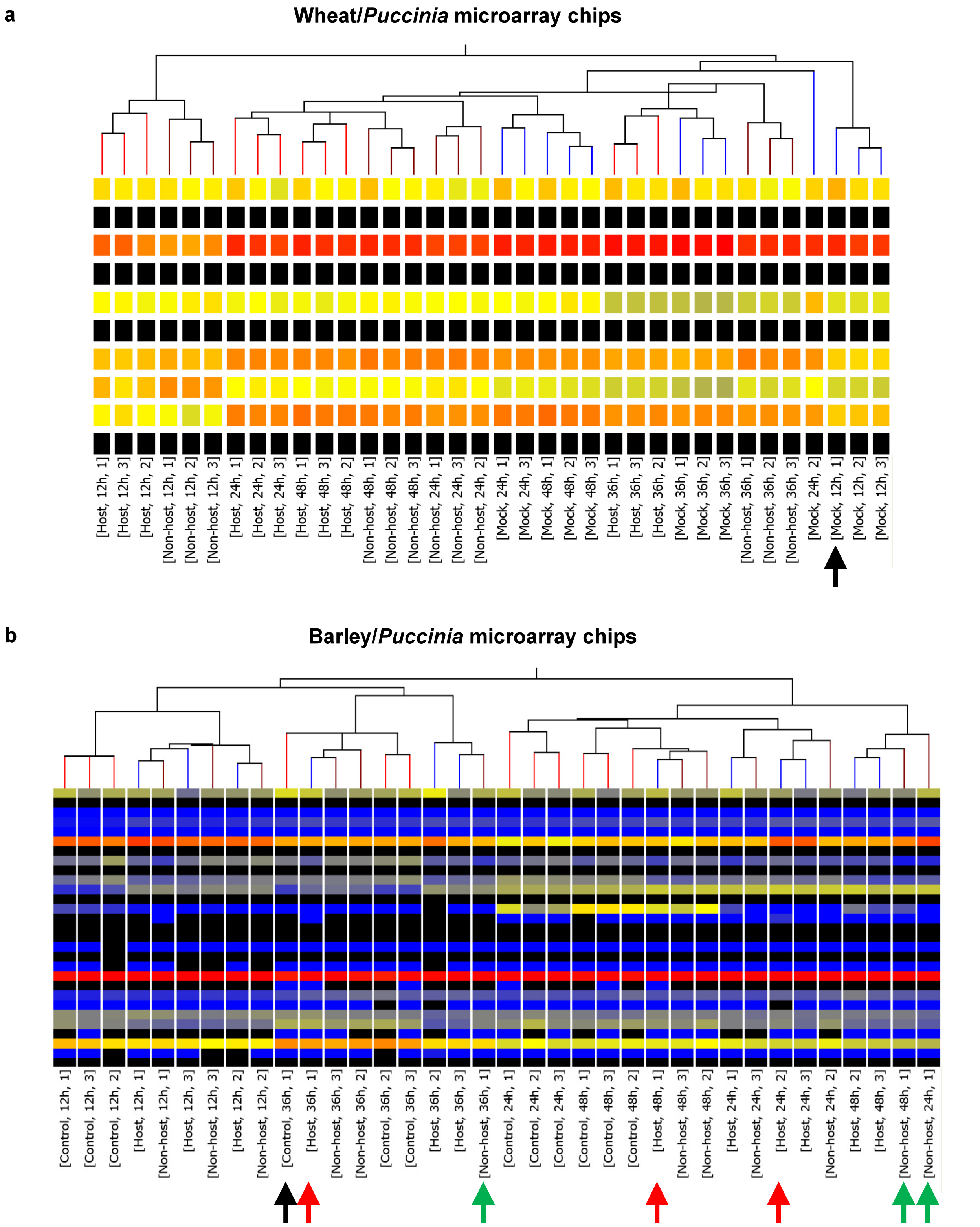

Figure S8. Clusterings of *Puccinia* microarray data leading to the decision to exclude one of three biological barley replicates. Ideally, the replications (1, 2, 3) from the same treatment (mock, host, nonhost) and time point (12, 24, 36 and 48 hpi) should cluster together. The clustering of chips was very clear in case of wheat/*Puccinia* interaction (**a**). In case of barley/*Puccinia* this was not the case: 7 out of 12 chips from replication 1 did not cluster into their respective subclades (**b**) (coloured arrows indicate chips out of their respective clusters). Therefore the complete replication 1 of barley/*Puccinia* interactions was omitted from the final analysis, resulting in a great enrichment of host/nonhost differentially regulated genes (11 according to analysis with all 3 replicates, 1824 according to analysis without replicate 1)
