# Supplementary material for: A comparative analysis of nonhost resistance across the two Triticeae crop species wheat and barley
Source: BMC Plant Biol. 2017 Dec 4;17:232. doi: 10.1186/s12870-017-1178-0 (PMC5715502; doi:10.1186/s12870-017-1178-0)
Supplement: Supplementary file 15 — Validation of microarray gene expression profiles by quantitative reverse transcription PCR (qRT-PCR). For selected candidate genes in the barley-Magnaporthe pathosystem, normalized signal intensities resulting from the microarray analyses (data points show averages and standard errors of three biological replicates) were compared with relative transcript abundancies resulting from qRT-PCR analysis of an independent experiment (bars show averages and standard deviations of two technical replicates). qRT-PCR was performed as described [69]. Asterisks indicate time points for which microarray analyses showed a significant differential expression during nonhost interaction compared to host interaction. In x-marked samples the respective candidate gene transcript was not detectable by qRT-PCR. (PDF 152 kb) [file 12870_2017_1178_MOESM15_ESM.pdf]

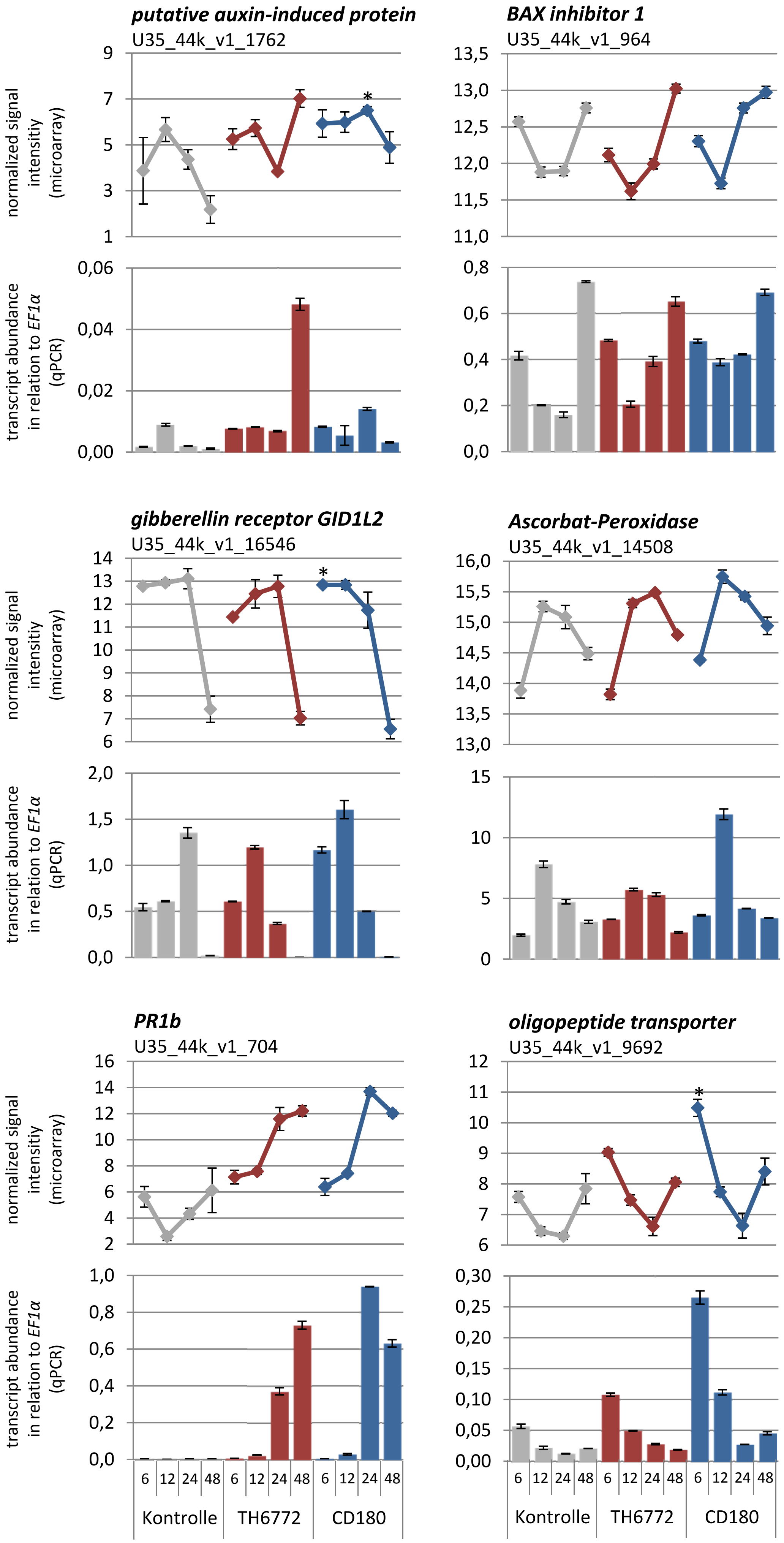

Figure S9. Validation of microarray gene expression profiles by quantitative reverse transcription PCR (qRT-PCR). For selected candidate genes in the barley-Magnaporthe pathosystem, normalized signal intensities resulting from the microarray analyses (data points show averages and standard errors of three biological replicates) were compared with relative transcript abundancies resulting from qPCR analysis of an independent experiment (bars show averages and standard deviations of two technical replicates). qPCR was performed as described [69]. Asterisks indicate time points for which microarray analyses showed a significant differential expression during nonhost interaction compared to host interaction. In x-marked samples the respective candidate gene transcript was not detectable by qPCR.
