# Supplementary material for: A comparative analysis of nonhost resistance across the two Triticeae crop species wheat and barley
Source: BMC Plant Biol. 2017 Dec 4;17:232. doi: 10.1186/s12870-017-1178-0 (PMC5715502; doi:10.1186/s12870-017-1178-0)
Supplement: Supplementary file 16 — Primer combinations used for validation of microarray gene expression profiles by qRT- PCR (Additional file 15: Figure S9). (PDF 21 kb) [file 12870_2017_1178_MOESM16_ESM.pdf]

Table S6. Primer combinations used for validation of microarray gene expression profiles by quantitative real-time PCR (Figure S9).

**gene (Organismus, ID)**	**forward primer**	**reverse primer**	**product**
ascorbate-peroxidase (*H. vulgare*, HT01F03Ta)	GAGTGGGGAGAAGGAAGGTC	TCATCCGCAGCATATTTGTC	99 bp
auxin-induziertes Protein (*H. vulgare*, U35 1762b)	ATAATTAGCGGAGGTGGCAGT	AAGAATAGGCAGCAAAAGCAA	100 bp
BAX Inhibitor 1 (*H. vulgare*, U35_964b)	ACCCTCTTCACCGACTTTGTT	CAGGACCTCCTCTTCCTCTTC	107 bp
GID1L2 (*H. vulgare*, U35_16546b)	AGTAGCAGTTGGCCGTTTCTT	ACACAGCCACTTCACCATAGC	128 bp
Oligopeptide-transporter, (*H. vulgare*, U35_9692b)	GCAAAGCATCTCCATGTGTGT	ATGACTGATTGCAGCACCATT	100 bp
PR1b (*H. vulgare*)	AACATCGTTGGACAGAAACCA	GGTGCATGAGATTAGGAACGA	98 bp
